# Breast and prostate cancers harbor common somatic copy number alterations that consistently differ by race and are associated with survival

**DOI:** 10.1186/s12920-020-00765-2

**Published:** 2020-08-20

**Authors:** Yalei Chen, Sudha M. Sadasivan, Ruicong She, Indrani Datta, Kanika Taneja, Dhananjay Chitale, Nilesh Gupta, Melissa B. Davis, Lisa A. Newman, Craig G. Rogers, Pamela L. Paris, Jia Li, Benjamin A. Rybicki, Albert M. Levin

**Affiliations:** 1 Department of Public Health Sciences, Henry Ford Health System, Detroit, MI USA; 2 Center for Bioinformatics, Henry Ford Health System, Detroit, MI USA; 3 Department of Pathology, Henry Ford Health System, Detroit, MI USA; 4 Center for the Study of Breast Cancer Subtypes, Breast Oncology Program, Department of Surgery, Weill Cornell Medical College, New York, NY USA; 5 Vattikuti Urologic Institute, Henry Ford Health System, Detroit, MI USA; 6 Department of Urology, Helen Diller Family Comprehensive Cancer Center, University of California at San Francisco, San Francisco, CA USA

**Keywords:** SCNAs, Race, Breast cancer, Prostate cancer, Genetic ancestry

## Abstract

**Background:**

Pan-cancer studies of somatic copy number alterations (SCNAs) have demonstrated common SCNA patterns across cancer types, but despite demonstrable differences in aggressiveness of some cancers by race, pan-cancer SCNA variation by race has not been explored. This study investigated a) racial differences in SCNAs in both breast and prostate cancer, b) the degree to which they are shared across cancers, and c) the impact of these shared, race-differentiated SCNAs on cancer survival.

**Methods:**

Utilizing data from The Cancer Genome Atlas (TCGA), SCNAs were identified using GISTIC 2.0, and in each tumor type, differences in SCNA magnitude between African Americans (AA) and European Americans (EA) were tested using linear regression. Unsupervised hierarchical clustering of the copy number of genes residing in race-differentiated SCNAs shared between tumor types was used to identify SCNA-defined patient groups, and Cox proportional hazards regression was used to test for association between those groups and overall/progression-free survival (PFS).

**Results:**

We identified SCNAs that differed by race in breast (*n* = 58 SCNAs; permutation *p* < 10^− 4^) and prostate tumors (*n* = 78 SCNAs; permutation *p* = 0.006). Six race-differentiated SCNAs common to breast and prostate found at chromosomes 5q11.2-q14.1, 5q15-q21.1, 8q21.11-q21.13, 8q21.3-q24.3, 11q22.3, and 13q12.3-q21.3 had consistent differences by race across both tumor types, and all six were of higher magnitude in AAs, with the chromosome 8q regions being the only amplifications. Higher magnitude copy number differences in AAs were also identified at two of these race-differentiated SCNAs in two additional hormonally-driven tumor types: endometrial (8q21.3-q24.3 and 13q12.3-q21.3) and ovarian (13q12.3-q21.3) cancers. Race differentiated SCNA-defined patient groups were significantly associated with survival differences in both cancer types, and these groups also differentiated within triple negative breast cancers based on PFS. While the frequency of the SCNA-defined patient groups differed by race, their effects on survival did not.

**Conclusions:**

This study identified race-differentiated SCNAs shared by two related cancers. The association of SCNA-defined patient groups with survival demonstrates the clinical significance of combinations of these race-differentiated genomic aberrations, and the higher frequency of these alterations in AA relative to EA patients may explain racial disparities in risk of aggressive breast and prostate cancer.

## Background

Both breast and prostate cancer exhibit racial disparities in incidence and outcomes that could be tied to underlying differences in genetics and disease biology. Relative to European American (EA) men, African American (AA) men are at a 1.7-fold increased risk of being diagnosed with prostate cancer and a 2.3-fold increased risk of dying from the disease [1]. While breast cancer incidence is slightly higher among EA women compared with AAs, mortality rates from this disease are more than 40% higher among AA women [1]. This mortality gap for AAs with breast cancer may be explained in part by the differential incidence of biologically-aggressive tumors that are negative for the estrogen receptor, the progesterone receptor, and the ERBB2 gene (commonly called triple negative breast cancer, TBNC), a phenotype that is twice as likely to occur in AA compared to EA women [2]. While molecular differences between these two racial groups have been investigated separately in both breast and prostate cancer, the degree to which somatic alterations that differ by race are shared across these two hormonally-driven cancers has not.

As breast and prostate cancer share some of the same biologic pathways, such as the steroid-hormone metabolism and insulin-like growth factor signaling, which when altered can lead to either cancer, a biologic rationale exists to investigate shared somatic genetic mechanisms between these two hormonally-driven cancers [3]. This concept of a shared somatic genetic etiology between breast and prostate cancer is supported by a recent study which used a gene expression panel, the PAM50, developed to molecularly subtype breast cancer, to classify prostate tumors into luminal- and basal-like subtypes [4]. Recent pan-cancer analyses have also shown breast and prostate cancer to have similar levels of insertion/deletion mutations [5] and intra-tumor heterogeneity [6], suggesting a similar mutational landscape may exist between the two cancers.

At a somatic genomic level, the mutational landscape of breast and prostate cancers are characterized by a large array of point mutations, but most tumors also demonstrate significant structural rearrangements of chromosomes and large segments of DNA gain and loss [7, 8]. These somatic copy number alterations (SCNAs) are associated with disease recurrence and survival in both breast [9–11] and prostate cancer [12–14]; however, these SCNA studies have included subjects principally of European ancestry. We have previously shown that SCNA biomarkers for aggressive prostate cancer established in EA populations may have utility in AAs [15], but the observed racial differences in SCNA frequencies in prostate cancer [16] suggest that larger somatic molecular studies of AAs are needed to better characterize these differences in frequency that may be related to disparities in disease outcome. Race-differentiated SCNAs have been shown to lead to gene expression differences in tumor immune response in prostate cancer [16] and associate with previously defined molecular subtypes in breast cancer [17].

While ethnic differences in SCNAs have been investigated before in both breast and prostate cancer, the datasets are typically small in size and with limited validation of the findings. Employing a novel approach that quantifies the magnitude of SCNA alteration, we used data from AAs and EAs in The Cancer Genome Atlas (TCGA) to identify SCNAs that 1) differ by race in breast and prostate cancer separately and 2) validate across these two hormonally- driven cancers. We further validated the race-differentiated SCNAs shared between breast and prostate cancer in two other hormonally driven cancers – endometrial and ovarian. We also tested the hypothesis that germline genetic African ancestry at an SCNA in TCGA admixed AAs (i.e. those whose genomes are comprised of ancestry from both Africa and Europe) could explain the observed differences in magnitude of SCNAs between AAs and EAs in both breast and prostate cancer. Finally, using an unsupervised approach, we evaluated whether underlying combinations of race-differentiated SCNAs defined patient groups that were associated with survival, and using gene expression data, we identified genes where expression was associated with both race and copy number within race-differentiated SCNAs.

## Methods

### Study subjects and molecular data

Data from The Cancer Genome Atlas (TCGA) were used for this study. TCGA somatic copy number (Affymetrix SNP 6 array) and germline genotype (Affymetrix SNP 6 array) data from the 22 autosomal chromosomes were downloaded from TCGA and are available in dbGaP, accession number phs000178.v11.p8 (https://ftp.ncbi.nlm.nih.gov/dbgap/studies/phs000178/phs000178.v11.p8/). These molecular data types were measured on 893 female breast cancer cases (719 EAs and 174 AAs) and 313 prostate cancer cases (270 EAs and 43 AAs). Self-reported AAs with a genome-wide African ancestry proportion (estimated as described in the Supplementary Methods) less than 20% and self-reported EAs with a genome-wide African ancestry proportion greater than 20% were removed from analysis. Samples with missing age-at-diagnosis and tumor severity data were also removed. After these quality control steps, 886 breast cancers and 309 prostate cancers remained, and the cancer specific characteristics are shown in Table 1.

**Table 1:**
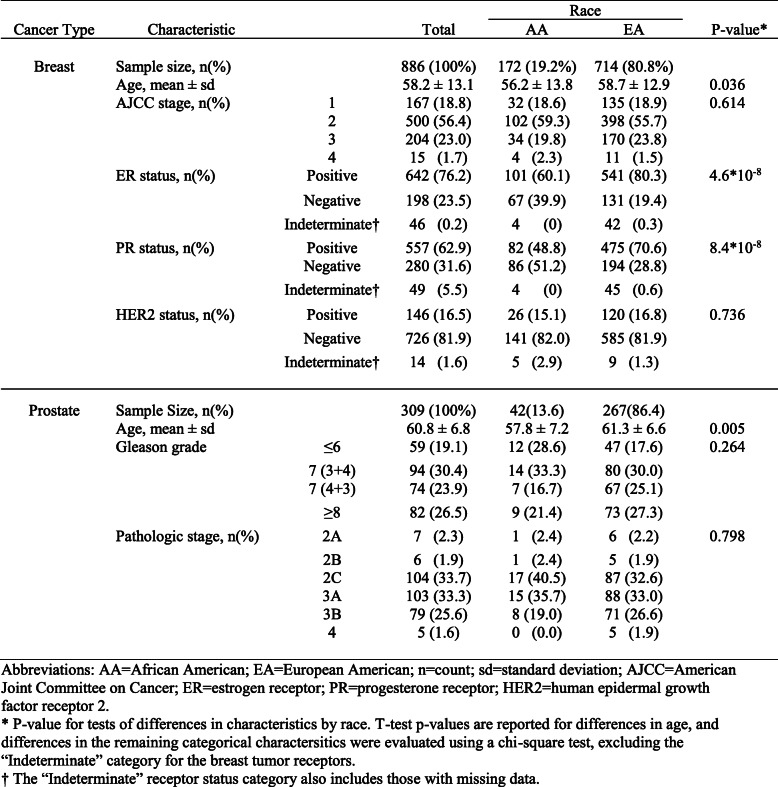
Characteristics of TCGA African American (AA) and European American (EA) breast and prostate tumors

### Statistical methods

Regional chromosomal ancestry was inferred using RFMix [18] (see Supplementary Methods). Genome-wide SCNA burden for each tumor was calculated as the percentage of the genome where the *log*_2_(*copy number*/2) differed (either positive or negative) from the tumor median by more than 0.1. Overall survival (OS) and progression-free survival (PFS) were downloaded from the pan-cancer TCGA publication [19] and events were censored at 10 years.

To identify commonly altered SCNAs, Gistic2 was utilized [20]. Briefly, Gistic2 models *log*_2_(*copy number*/2) at each SNP location across tumor genomes and identifies frequent SCNAs through the use of both the frequency and quantitative degree of a given alteration in the sample of tumors being analyzed. For each tumor type, SCNAs were identified using Gistic2 in AAs and EAs separately to account for the differences in samples size (i.e. > 3 times more EAs in both tumor types). The resulting non-identical SCNA boundaries between AAs and EAs were resolved such that a partially overlapping region could be divided into as many as three sub-regions based on the overlapping status: identified only in AA, identified only in EA, or identified in both groups. The approach to the reconciliation of the degree of overlap between SCNAs between the two groups is detailed in the Supplementary Methods and Figure S1.

To identify SCNAs that differ between AA and EA individuals, we quantified the copy number magnitude across each SCNA by calculating the area under the copy number curve (cnAUC) (See Supplementary Methods and Figure S2). For this measure, positive area (above the null value of two copies) corresponds to amplification and negative area (below the null value of two copies) corresponds to deletion.

To assess the association between cnAUC and race for each SCNA, the following linear model was used:

$$ cnAUC= race+ age+ tumor\ pathology $$

For the dichotomous race variable, EA race was treated as the referent group. As such, a positive race difference in amplified SCNAs and a negative race difference in loss SCNAs represent higher magnitude copy number amplifications and deletions, respectively, in AA relative to EA tumors. These models were also adjusted for age at diagnosis and tumor pathology (pathological stage for the breast, endometrial, ovarian, lung and kidney cancer analyses; and Gleason grade for the prostate cancer analyses). To be inclusive and account for the lower number of AA prostate tumors (*n* = 42) relative to AA breast tumors (*n* = 172), a *p*-value threshold of 0.1 was used to identify potential SCNAs with differences between races. To allow the comparison of race coefficients of SCNAs with different lengths, the coefficients were standardized by each SCNA’s length. To assess the statistical significance of the total number of race-differentiated SCNAs observed both within each tumor type and shared between tumor types, permutation tests were performed, with details presented in Supplementary Methods. A similar linear model was used to quantify the relationship between SCNAs and regional ancestry (see Supplementary Methods)

To identify underlying patient groups based on the race-differentiated SCNAs shared by both breast and prostate cancer, unsupervised hierarchical clustering, using the complete linkage algorithm and Euclidean distance, was applied to the *log*_2_(*copy number*/2) of genes residing in the six consistently race-differentiated SCNAs. The gap statistic [21] was used to determine the optimal number of clusters, where the optimal number of clusters, *k*, is the first *k* where *k* + 1 clusters does not lead to an increase of the gap statistic. Three patient groups in both breast and prostate cancer were determined to be optimal and were visualized using a copy number-based heatmap. Chi-square tests were used to assess the association between patient group and race or TNBC status, and log-rank tests were used to test for differences in Kaplan-Meier survival curves among different groups. Cox proportional hazards models were used to test the association of patient group with 10-year OS and PFS while adjusting for race, age-at-diagnosis, tumor pathology, and SCNA burden.

$$ survival= patientgroup+ race+ age+ tumor\ pathology+ SCNA. burden $$

To investigate differences in transcriptional gene expression within the six consistently race-differentiated SCNAs, the raw, gene-level mapped Illumina RNASeqV2 sequencing reads were analyzed using the R *limma* package [22]. Briefly, the raw read counts were normalized by total number of mapped reads in each sample, yielding counts per million (cpm),and further log transformed (log-cpm). The log-cpm values allow for the use of standard linear regression modeling to identify differentially expressed genes by race. Age-at-diagnosis, tumor pathology (pathological stage for breast cancer and Gleason grade for prostate cancer) were also included in the model. The Benjamini-Hochberg false discovery rate (FDR) was used to adjust *p*-values for multiple comparisons. For those genes with significant differential expression by race, we also assessed whether copy number at the gene-level was associated with gene expression using linear regression.

## Results

### SCNAs in TCGA breast tumors and differences by race

**Fig. 1:**
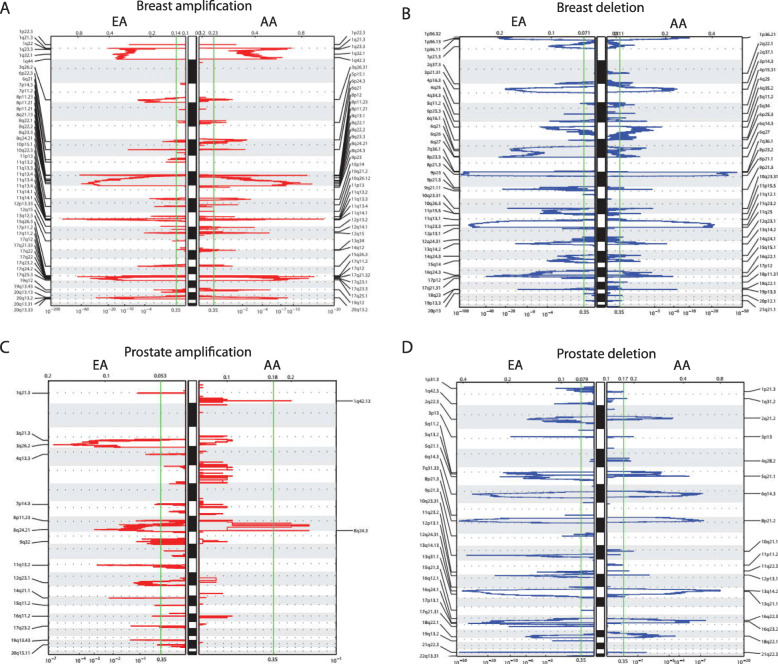
Autosomal SCNAs identified by Gistic2. **a** Breast tumor amplification SCNAs in European American (EA) and African American (AA) breast tumors. **b** Breast tumor deletion SCNAs in EAs and AAs. **c** Prostate tumor amplification SCNAs in EAs and AAs. **d** Prostate tumor deletion SCNAs in EAs and AAs. The autosomes are arranged in chromosomal order from top to bottom, and the horizontal dotted lines indicate the centromere for each chromosome. The cytobands are plotted on the left and right of each panel only for significant SCNAs. The bottom axis shows the false discovery rate (FDR) for SCNA detection, and the green line indicates the default significance threshold (FDR < 0.35) used by Gistic2 to identify SCNAs

Given the higher frequency of tumor data for EAs in comparison to AAs in TCGA breast and prostate tumor sets, we elected to identify SCNAs separately by race group in order not to obscure SCNAs that might be specific to AAs. From the 714 EA TCGA breast tumors (Table 1), Gistic2 identified 55 SCNAs (Fig. 1a and b). Of these, 24 (43.6%) were amplifications, and 31 (54.6%) were deletions. In the 172 AA TCGA breast tumors (Table 1), 57 SCNAs were identified (Fig. 1a and b). Of these, 27 (47.4%) were amplifications, and 30 (52.6%) were deletions. To integrate SCNAs identified separately in EA and AA breast tumors, non-overlapping SCNAs between the two groups were retained as is, while overlapping SCNAs were sub-divided based on the degree of overlap (see Supplementary Methods and Figure S1). This integration resulted in a total of 132 unique SCNAs.

Overall, AA breast tumors had a higher percentage of their genomes affected by SCNAs (termed “SCNA burden”; average 47.5%) in comparison to EAs (39.6%; *p* = 3*10^− 4^; Figure S3). To assess specific regional SCNA differences between AA and EA tumors, the degree of alteration at each SCNA was quantified as the area under the copy number (${\mathit{\log}}_2\frac{copy\ number}{2}$) curve (cnAUC) for each patient (see Methods, Supplementary Methods, and Figure S2). For these analyses, positive race differences in amplified SCNAs (i.e. AA_cnAUCgain_-EA_cnAUCgain_ > 0) and negative race differences in deletion SCNAs (i.e. AA_cnAUCloss_-EA_cnAUCloss_ < 0) represent higher magnitude copy number gains and losses in AA relative to EA tumors, respectively. In breast tumors, there were 58 SCNAs (44% of 132) that showed significant differences by race (individual SCNA *p* < 0.1; Table S1), and this total number of breast cancer race-differentiated SCNAs was greater than what would be expected by chance alone (permutation *p* < 10^− 4^; see Supplementary Methods and Figure S4). Of these 58 SCNAs, 25 were amplifications and 33 were deletions. For the majority of both amplifications and deletions, AA breast tumors had higher magnitude SCNAs relative to their EA counterparts. Specifically, of the 25 amplifications, 18 (72%) had positive differences (i.e. more gain in AA tumors), and in the 33 deletions, 22 (66.7%) had negative differences (i.e. more loss in AA tumors). In particular, for the top five most significant race-differentiated SCNAs, all had higher magnitude copy number changes in AAs, and these SCNAs fell into the following two loci: two adjacent deletions on chromosome 5 (5q11-5q14, *p* = 3.48*10^− 6^; 5q14-5q22, *p* = 1.57*10^− 5^) and three contiguous amplifications on chromosome 10 (10p15, *p* = 7.24*10^− 5^; 10p15, *p* = 9.92*10^− 5^; 10p15–10p13, *p* = 1.87*10^− 5^).

### SCNAs in TCGA prostate tumors and differences by race

Overall, AA prostate tumors had a higher SCNA burden (average 32.3%) than EA prostate tumors (average 18.5%; *p* = 0.028; Figure S3). In the 267 EA TCGA prostate tumors (Table 1), Gistic2 identified 43 SCNAs (Fig. 1c and d). Of these, 17 (39.5%) were amplifications, and 26 (60.5%) were deletions. In the 42 AA TCGA prostate tumors (Table 1), 22 SCNAs were identified (Fig. 1c and d). Of these, two were amplifications (9.1%), and 20 were deletions (90.9%). These SCNAs were pooled in the same manner as the breast cancer results (see Supplementary Methods), which resulted in 74 unique prostate cancer SCNAs.

A total of 21 SCNAs (28% of 74; 5 amplifications and 16 deletions) significantly differed by race (*p* < 0.1, Table S2), and this total number of prostate cancer race-differentiated SCNAs was greater than what would be expected by chance alone (permutation *p* = 0.0064; see Supplementary Methods and Figure S4). Similar to the breast tumors, AA prostate tumors displayed a greater percentage of higher magnitude SCNAs at both amplifications (80%) and deletions (81%).

### Race-differentiated SCNAs common to both breast and prostate tumors and the impact of germline African ancestry

**Fig. 2:**
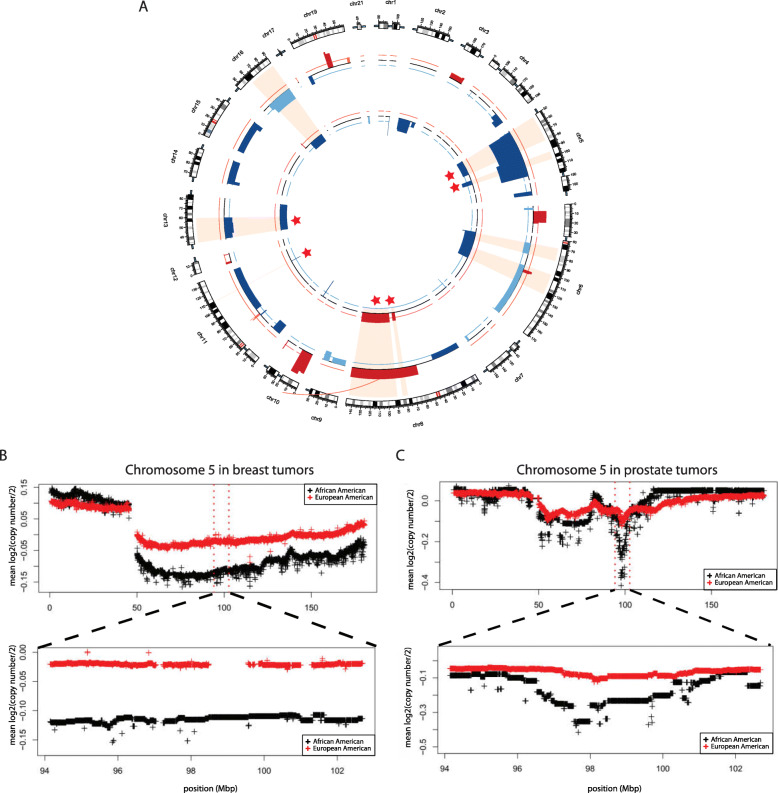
Race-differentiated SCNAs identified in TCGA breast and prostate tumors. **a** Circos plot of all race-differentiated SCNAs (*p* < 0.1) identified in TCGA breast (outer circle) and prostate (inner circle) tumors. The red color indicates SCNAs with higher magnitude amplification in African Americans (AAs), and the orange color indicates SCNAs with higher magnitude amplification in European Americans (EAs). The dark blue color indicates SCNAs with higher magnitude deletions in AAs, while the light blue shows SCNAs with higher magnitude deletions in EAs. The nine shaded regions indicate alterations that exists in both tumor types. Among those nine, the six SCNAs with consistent differences by race across tumor types are indicated by stars. As an example of one consistent region, average log_2_ (copy number/2) profiles on chromosome 5 were plotted for each race in **b** breast and **c** prostate tumors. For each tumor type, the top panel corresponds to the complete chromosome 5 profile, and the lower panel corresponds to chromosome 5q15–21.1, where the actual boundaries are from the prostate tumor SCNA

Next, we compared the race-differentiated SCNAs across these two tumor types. A total of nine (42%, 9 of 21) race-differentiated SCNAs identified in prostate tumors overlapped with race-differentiated breast cancer SCNAs (Fig. 2a). These nine SCNAs reside on chromosomes 5q11.2-q14.1, 5q15-q21.1, 6q12-q14.2, 6q16.2–22.31, 8q21.11-q21.13, 8q21.3-q24.3, 11q22.3, 13q12.3–21.3, and 16q21-q24.3. In both tumor types, the chromosome 8 SCNAs were the sole amplifications, and the remaining seven were deletions. For six of the nine (67%) overlapping SCNAs, the direction of the race difference was consistent in both cancer types, which was more than what would be expected by chance alone (permutation *p* = 7*10^− 4^; see Supplementary Methods and Figure S4). For all six alterations, AA prostate and breast tumors both had higher magnitude alterations relative to EAs. These included two amplification SCNAs on 8q21.11-q21.13 and 8q21.3-q24.3 and four deletion SCNAs on 5q11.2-q14.1, 5q15-q21.1, 11q22.3, and 13q12.3–21.3 (Fig. 2a). Figure 2b and c display the chromosome 5q15-q21.1 race-differentiated SCNAs average cnAUC profiles in both breast and prostate tumors, respectively. Similar plots for the remaining four SCNAs are presented in Figure S5, Figure S6, Figure S7, and Figure S8.

To further validate the six race-differentiated SCNAs, we tested for racial differences in the following four additional tumor types with sufficient data for AAs in TCGA: endometrial, ovarian, lung, and kidney tumors. The results of the SCNA differences by race for these four tumor types are presented in Table S3. From the six alterations and consistent with the breast and prostate results, the amplification SCNA on 8q21.3-q24.3 and deletion SCNA on 13q12.3–21.3 were more extreme in AAs relative to EAs in endometrial cancer; this was also the case with the deletion SCNA on 13q12.3–21.3 in ovarian cancer (Table S3).

As AAs are admixed (i.e. have genomes composed of DNA from more than one ancestral population, African and European ancestral populations for AAs), we investigated whether germline chromosomal ancestry (either African or European) differences at each SCNA can explain the greater magnitude of SCNAs observed in admixed AA tumors. A consistent association with local ancestry in these SCNAs would be indicated by: 1) a positive difference in amplification SCNAs reflecting increasing gain associated with increasing regional chromosomal African ancestry or; 2) a negative difference in deletion SCNAs reflecting more loss associated with increasing regional chromosomal African ancestry. By these criteria, the amplification at 8q21.3-8q24.3 and the deletion at 13q12.3-13q21.3 in both breast and prostate tumors showed African ancestry differences consistent in direction with the race association analyses, but these did not reach statistical significance in either tumor type (Table S4).

### Unsupervised hierarchical clustering using copy number of genes residing within the six race-differentiated SCNAs identified patient groups with differences in survival

**Fig. 3:**
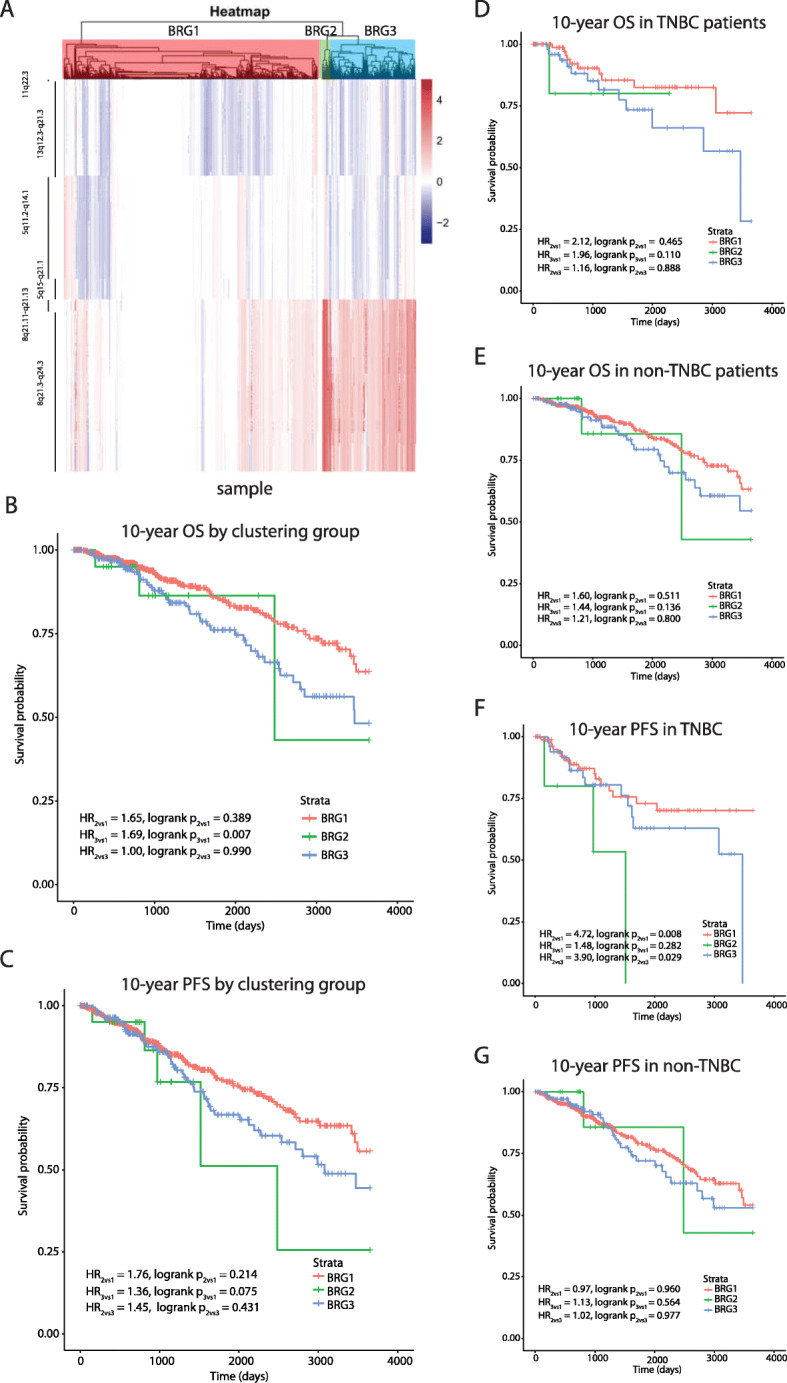
Hierarchical clustering identified patient groups based on race-differentiated SCNAs in breast tumors and their association with survival. **a** Hierarchical clustering and the gap statistic identified three molecular breast cancer patient groups (BRG1–3) based on race-differentiated SCNAs shared with prostate cancer, and the contributions of the component SCNA to each are displayed in the SCNA heatmap. **b** Kaplan-Meier 10-year overall survival curves by BRG. **c** 10-year progression-free survival (PFS) curves by BRG. Kaplan-Meier 10-year overall survival (OS) curves by BRG for women **d** with triple negative breast cancer (TNBC) and **e** those without TNBC. Kaplan-Meier 10-year PFS curves by BRG for women **f** with TNBC and **g** those without TNBC

To assess the clinical relevance of the six race-differentiated SCNAs consistent across both breast and prostate cancer, unsupervised hierarchical clustering was first used to classify samples based on the copy number of genes residing within these alteration regions in breast and prostate cancer separately. In breast cancer, three patient groups (labelled as BRG1–3) were identified (Fig. 3a) based on the gap statistic [21] (Figure S9A), and BRG2 and 3 contained tumors with more copy number changes. Consistently, AAs were more likely classified as BRG2 and BRG3 (Table S5, *p* = 0.01). The three patient groups also exhibited differences in both 10-year overall survival (OS) and 10-year PFS, with BRG2 and 3 group patients having worse OS (Fig. 3b; hazard ratio [HR] HR_BRG3vs1_ = 1.69, *p* = 0.007; HR_BRG2vs1_ = 1.65, *p* = 0.389) and PFS (Fig. 3c; HR_BRG3vs1_ = 1.36, *p* = 0.075; HR_BRG2vs1_ = 1.76, *p* = 0.214) relative to BRG1. In a multivariate model, adjusting for race, age, pathologic stage, and SCNA burden, BRG3 had poorer survival relative to BRG1 (Table 2; OS HR_BRG3vs1_ = 1.72, p = 0.01; and PFS HR_BRG3vs1_ = 1.34, *p* = 0.117). Interestingly, no significant differences were observed between AA and EA breast tumors by BRG for 10-year OS (race interaction *p*-value = 0.393) or PFS (race interaction *p*-value = 0.347; Figure S10).

**Table 2:**
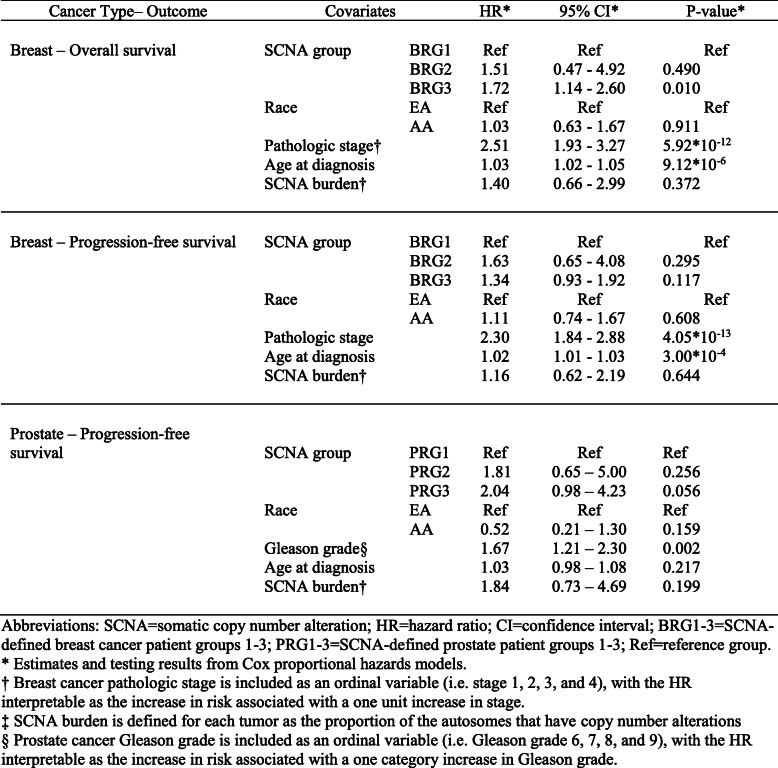
Association* of SCNA defined patient groups with breast and prostate cancer survival

Compared with BRG1, BRG2 and BRG3 patients were more likely to have triple negative breast cancer (TNBC) (Table S5; *p* = 0.003). Among TNBCs, BRG2 patients demonstrated worse PFS relative to both BRG1 (Fig. 3f, HR_BRG2vs1_ = 4.72, *p* = 0.008) and BRG3 (HR_BRG2vs3_ = 3.9, *p* = 0.029). Further, relative to BRG1, BRG3 patients had worse OS (Fig. 3d, HR_BRG3vs1_ = 1.96, *p* = 0.110) and worse PFS (Fig. 3f, HR_BRG3vs1_ = 1.48, *p* = 0.282), although these differences did not reach statistical significance. The BRGs did not further stratify survival risk among non-TNBC patients based on OS (Fig. 3e) or PFS (Fig. 3g).

**Fig. 4:**
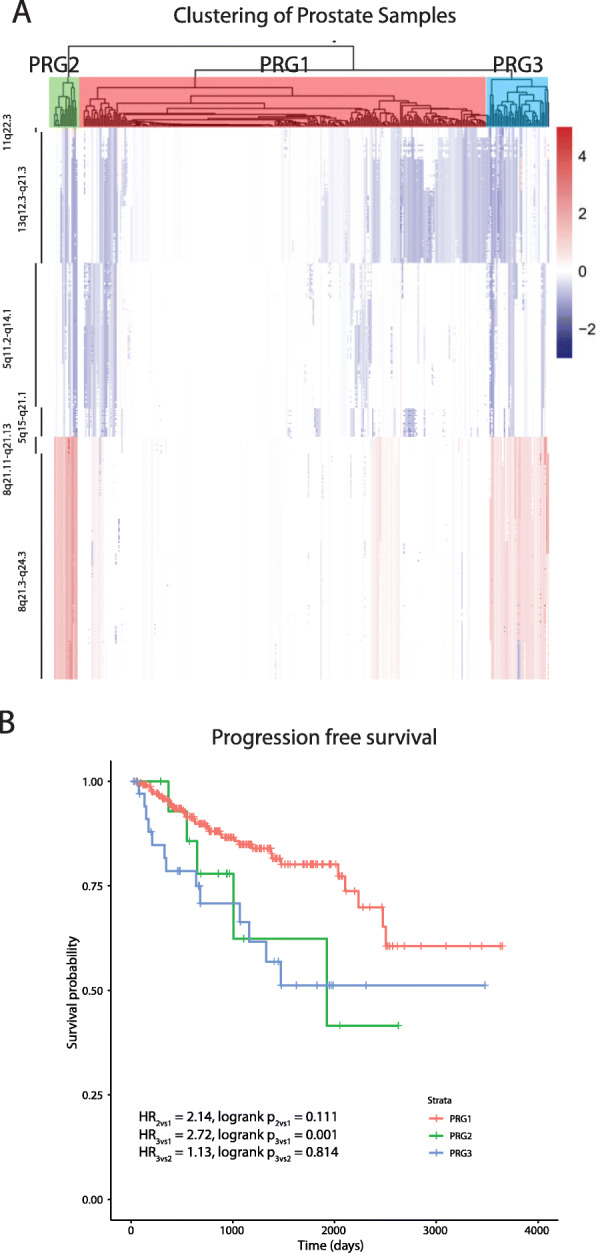
Hierarchical clustering identified patient groups based on race-differentiated SCNAs in prostate tumors and their association with 10-year PFS. **a** Hierarchical clustering and the gap statistic identified three molecular prostate cancer patient groups (PRG1–3) based on race-differentiated SCNAs shared with breast cancer, and the contributions of the component SCNA to each are displayed in the SCNA heatmap. **b** Kaplan-Meier 10-year Progression Free Survival (PFS) curves by PRG

For prostate cancer, three patient groups were identified (Figure S9B) and labelled as PRG1–3 (Fig. 4a). AAs appeared more frequently in PRG2 (Table S5; *p* = 0.069). Due to the limited number of OS events, analyses were restricted to10-year PFS. PRG2 and PRG3 patients demonstrated worse 10-year PFS compared with PRG1 (Fig. 4b, HR_PRG2vs1_ = 2.14, *p*-value = 0.111; HR_PRG3vs1_ = 2.72, *p*-value = 0.001). In a multivariate model, adjusting for race, age, Gleason grade, and SCNA burden, PRG3 patients had suggestive worse survival relative to PRG1 patients (Table 2, HR_PRG3vs1_ = 2.04, *p* = 0.056). Consistent with the breast cancer results, there was no evidence that the association between PRGs and PFS differed between AAs and EAs (race interaction *p*-value = 0.977; Figure S11).

### Differentially expressed genes in the six race-differentiated SCNAs common to breast and prostate cancer

To identify race-differences in gene expression consistent with the differences in copy number magnitude within the six race-differentiated SCNAs in both breast and prostate tumors, we performed differential gene expression analyses in both tumor types using TCGA RNA-Seq expression data. Of 522 unique mRNA transcripts in theses six race-differentiated SCNAs, after adjusting for multiple comparisons using a false discovery rate (FDR) of 5%, a total of 18 mRNAs (Table S6) were consistently differentially expressed by race in both tumors. Among these 18 genes, we further assessed whether copy number was associated with gene expression, and the results are summarized in Table S7. We identified 15 genes in breast cancer and 16 genes in prostate cancer where gene expression was significantly (FDR < 0.05) associated with copy number, and 15 of these were shared between breast and prostate cancer. Multiple cancer related genes were represented in this list including *TOP1MT* (mitochondrial topoisomerase I), *GLI4* (glioma-associated oncogene homolog 4), *RB1* (retinoblastoma), *KBTBD7* (Kelch Repeat and BTB Domain Containing 7), *KBTBD6* (Kelch Repeat and BTB Domain Containing 6), *MARVELD2* (MARVEL Domain Containing 2, also called Tricellulin), *OCLN* (Occludin), and *PVT1* (Pvt1 oncogene).

## Discussion

Breast and prostate cancer are two hormonally-driven cancers with established racial disparities in both incidence and severity in women and men, respectively. SCNA events can be considered a primary driver of carcinogenesis across these two cancers, and in our analysis of TCGA breast and prostate cancer copy number alteration data, we demonstrated that a majority of the race-differentiated SCNAs for either tumor type was of greater magnitude in AAs. Further, inter-tumor comparisons demonstrated six SCNAs common to both cancers that had higher magnitude SCNAs in AAs. Two of these race-differentiated SCNA were validated in endometrial and ovarian cancer, two other hormonally-driven cancers. Using unsupervised hierarchical clustering on copy number of genes residing in these six SCNAs, we demonstrated three patient groups in breast cancer and three patient groups in prostate cancer with distinct survival profiles, and these patient groups further differentiated within TNBC based on PFS. Finally, within these six race-differentiated SCNAs shared by both breast and prostate tumors, 15 mRNA transcripts were differentially expressed by race and copy number in both tumors types, consistent with observed SCNA differences, and these included known cancer genes.

AA women with breast cancer may have greater intra-tumor genetic heterogeneity and increased basal gene expression even within the triple-negative phenotype, suggesting a more aggressive tumor biology among AA patients [23]. However, a recent analysis of TCGA data on 178 TNBCs found no difference in somatic mutation counts by race [24], which suggests that structural DNA changes may play a greater role in driving racial tumor biology differences. In our current analysis of TCGA breast tumors, 69% of the SCNAs identified were of greater magnitude in AAs. A previous study [17] of 53 AA and 206 EA breast tumors using array comparative genomic hybridization (aCGH) with 4320 probes showed nine race-differentiated SCNAs in triple-negative cases on chromosomes 5q, 8q, 9q, 10q, 14q and 15q, where AAs had greater changes in all nine SCNAs. Four (5q31, 8q21–24, 14q32, and 15q12) of these overlapped with regions identified in our analyses.

In a recent analysis of TCGA breast cancer data by Hou et al. [25], AAs had more loss in four regions (8p23, 13q14, 11q23, and 21q21) and more gains in six regions (8q24, 8p12, 8q11, 19q12, 5p15, and 12q15) based on analysis of nominal copy number deletion and amplification calls. Using our magnitude-based approach (i.e. cnAUC), which additionally adjusted for age at diagnosis and pathological stage, loss on 8p23, 13q14, 11q23 and gains on 8q24, 8q11 and 19q12 were greater in AAs similar to Hou et al. We did not identify race-differentiated SNCAs in the other four regions, but we did identify additional race-differentiated SCNAs (19 deletions and 11 amplification; Fig. 1a and b and Table S1).

While our breast cancer analyses utilized a similar TCGA tumor dataset as Huo et al. [25], there are distinctions between the approaches that may have led to differences in our findings beyond the slight difference in the number of breast tumors available at the time of analysis. As mentioned above, Huo et al. discretized SCNAs into nominal gains and losses, and their analysis compared the differences in the frequency of SCNAs between the two races. In comparison, we used a novel quantitative measure across each SCNA for each tumor, which not only captures differences in SCNA frequency but also incorporates the alteration magnitude. As the use of this additional information identified more race-differentiated SCNAs relative to Huo et al. [25], our approach may be more sensitive to the detection of such differences. Another important distinction is subject inclusion. While both studies utilized the germline genetic data available to estimate genetic ancestry and the agreement between ancestry and self-reported race to select subjects, we applied a more stringent threshold for study entry. For example, Huo et al. included 86 subjects with missing self-reported race data by assigning them an ethnicity most consistent with their genetic ancestry. In contrast, we chose to only include subjects whose self-reported race agreed with their genetic ancestry. Our approach is likely more conservative, which again may impact differences in findings. Finally, Hou et al. did not adjust for pathologic stage in their analysis, which is correlated with the SCNAs. Not accounting for pathologic stage may confound the relationship between SCNAs and race, potentially producing a false positive effect that is simply due to differences in pathologic stage between AAs and EAs.

In addition to breast cancer, growing evidence also suggests that prostate tumors of AA men undergo somatic changes that are differ from EA men. For example, Khani et al. [26] showed that tumors of AA men have less *ERG* rearrangement and *PTEN* deletion but more *SPINK1* overexpression than clinically similar tumors of EA men. In prostate tumors, we identified five amplification and 16 deletion SCNAs that differed by race. Among these SCNAs, loss at 6q13–22, 13q13–14, and 16q11–24, and gains of 8q24 also showed higher frequency in 20 AA prostate tumors from a previous genome-wide SCNA scan [27]. In another study [16] where the SCNAs of 28 AA and 180 EA prostate tumors were profiled by aCGH, 23 regions were identified as race-differentiated, and in 21 (91.3%) regions, AA tumors had more copy number changes. The greater frequency of deletions in AA tumors observed on chromosomes 1q31 and 16q22 were consistent with our findings. Combined with the literature on race-differentiated SCNAs in breast tumors, these studies collectively suggest that the genomes of AA breast and prostate tumors are less stable and harbor greater numbers of SCNAs compared with their EA counterparts.

While the mechanism(s) giving rise to this difference in stability are unknown, one possible explanation is differential expression of common fragile sites (CFS). CFS expression is known to give rise to SCNAs, including deletions that lead to tumor suppressor gene deactivation in multiple cancer types [26], and currently, there are 125 known CFS spread throughout the human genome. Using CFS genomic coordinates provided in the HumCFS database [28], we found that four of the six race-differentiated SCNAs common to both breast and prostate cancer overlapped with CFS (5q11.2-5q14.1 with FRA5H, 5q15-5q21.1 with FRA5B and FRA5F, 8q21.3-8q24.3 with FRA8D, 13q12.3-13q21.3 with FRA13B and FRA13C), including those that were also observed in endometrial (8q21.3-q24.3 and 13q12.3-q21.3) and ovarian (13q12.3-q21.3) cancers. This observation raises the provocative question of whether CFS expression in tumors differs by race of the individual. While evaluating CFS expression as a possible mechanism underlying race-differentiated SCNAs is outside of the scope of this paper, our observation suggests that it is worthy of further investigation.

Using the gene-level copy number information within the six race-differentiated SCNAs that have consistent directional differences by race across both tumor types, we identified three patient groups in both breast and prostate tumors through unsupervised hierarchical clustering. Groups containing samples with more frequent copy number changes in these six regions are associated with poorer OS and PFS in breast cancer and worse PFS in prostate cancer. These differences remain after adjusting for self-reported race, age-at-diagnosis, tumor pathology, and genome-wide SCNA burden, highlighting the clinical significance of identified SCNA patient groups. Poorer breast and prostate cancer outcomes in AAs compared to EAs is at least partly explained by the higher incidence rates of biologically aggressive disease in AAs. In breast cancer, AAs had a two-fold higher incidence rates of TNBC compared with EAs [29], and evidence suggests that prostate cancer progresses more rapidly for AA men, who experience a higher prostate cancer volume at diagnosis and a four-times higher incidence of metastatic disease relative to EA men [30]. A previous analysis of TCGA data reveled that AA TNBC patients had a shorter time to disease progression compared with EAs, but no disparity with hormone receptor-positive or HER2/neu-positive patients [23]. Other studies have demonstrated similar rates of disease progression for AA and EA TNBC patients but poorer survival for AA cases with hormone receptor positive tumors [23, 31, 32]. Our analyses examining the effect of race within each identified SCNA group demonstrated a lack of statistically significant differences in survival between AAs and EAs. This observation favors the hypothesis that breast and prostate cancer outcome disparity between AA and EA is due to a higher frequency of deleterious SCNAs in AA relative to EA tumors. Furthermore, we demonstrated that in TNBC and hormone receptor-positive breast cancer, survival differences still exists between SCNA-defined groups, suggesting that these patient groups may provide additional prognostic information to currently defined tumor subtypes. Independent studies will be needed to confirm the association between the SCNA patient groups we have identified and survival.

Within the six SCNAs that were found to have consistent differences in magnitude by race across breast and prostate tumors, we identified 15 genes that were differentially expressed by race and copy number, many of which have known cancer related functions. For example, *TOP1MT* on 8q24.3, a mitochondrial DNA topoisomerase, was the gene with the most significant difference in expression by race in both tumor types. *TOP1MT* has c-myc binding sites in its promoter and follows the expression of the master regulator oncogene, c-myc [33]. The well-characterized tumor suppressor gene *RB1* (retinoblastoma susceptibility gene) on 13q14.2 displayed significantly lower expression in AA breast and prostate tumors, which correlates with the greater loss of DNA we observed in AAs relative to EAs. *RB1* was the first molecularly defined tumor suppressor by suppressing cell cycle progression and controlling chromatin remodeling and cell death [34, 35]. *RB1* loss is associated with tumor progression in both breast and prostate cancers [36–38]. The long non-coding RNA *PVT1* within the amplification SCNA identified on 8q24.21 is overexpressed in both AA breast and prostate tumors, and it was found that the overexpression of the *MYC* oncogene in tumors depends on *PVT1* expression [39]. In summary, these findings suggest that genes known to be involved in tumorigenesis and cancer progression, are similarly differentially expressed by race and copy number and may, therefore, underlie racial differences in tumor biology linked to the six race-differentiated SCNAs between AA and EA breast and prostate tumors.

There are multiple limitations in this study that should be mentioned. First and foremost is the under-representation of AAs (and other minorities) in TCGA relative to EAs. An important consequence of this reality is the limitation in statistical power to detect underlying racial differences in SCNAs both within and across tumor types. While we were able to identify statistically significant aggregate racial differences in SCNAs using a permutation approach, there are likely additional, potentially more subtle racial differences in SCNAs that were missed in this analysis. Further, our ability to explore how these differences were shared across different tumor types was similarly curtailed by this limitation. Also, while we were able to adjust our analyses for important variables that could confound both the relationship between race and SCNAs and the relationship between SCNAs and survival, this list of potential confounders was limited in TCGA. Together, coupled with the positive findings from this study, these limitations suggest that larger genomic studies with racial balance and comprehensive and consistent clinical/pathologic data collection are necessary to make further progress towards understanding how race influences cancer biology and outcomes.

## Conclusions

In summary, using TCGA data, we demonstrated that a majority of race-differentiated SCNAs in breast and prostate tumors have greater magnitude alterations in AAs relative to EAs and that this was the case for all of six race-differentiated SCNAs that were shared consistently across these tumor types. Combined, these findings suggest that breast and prostate tumor genomes of AAs may be more susceptible to SCNAs that underlie a more aggressive phenotype. This information may be useful for deciding whether adjuvant treatment is needed after surgery in the subset of patients with SCNAs associated with worse outcomes. Finally, while this study focused on breast and prostate tumors, our additional findings from endometrial and ovarian tumors that are consistent with a subset of the racially differentiated SCNAs in breast and prostate cancers suggest that this genomic phenomenon may be representative of other tumor types with racially disparate outcomes, which warrants further studies with appropriate race representation.

## Supplementary information

**Additional file 1. **Supplementary Methods. Pdf format. The document describes the following methods used in the analysis: estimating local ancestry and validating self-reported race; identifying recurrent SCNAs through Gistic2; integration of SCNA regions identified by Gistic2 in African American (AA) and European American (EA) tumors separately; calculation of SCNA magnitude for each tumor using an area under the copy number curve approach; assessment for multiple testing using a permutation approach; and association of local ancestry with race-differentiated SCNAs.

**Additional file 2: Table S1.** Pdf format. Somatic copy number alterations (SCNAs) in TCGA breast tumors (*n* = 886) that differ significantly by race. 58 SCNAs that showed significant differences by race in breast tumors are listed in this table along with information on their chromosomal location, length, SCNA type (deletion or amplification), cytoband, beta coefficient, standard error, test statistic, and *p*-value.

**Additional file 3: Table S2.** Pdf format. SCNAs in TCGA prostate tumors (*n* = 309) that differ significantly by race. 21 SCNAs that showed significant differences by race in prostate tumors are listed in this table along with information on their chromosomal location, length, SCNA type (deletion or amplification), cytoband, beta coefficient, standard error, test statistic, and *p*-value.

**Additional file 4 Table S3**. Pdf format. Additional cross-tumor validation of the six race-differentiated SCNAs in TCGA.

**Additional file 5: Table S4.** Pdf format. Association of regional chromosomal African ancestry with SCNAs among African Americans in the six regions where African Americans had higher magnitude SCNAs relative to European Americans in both prostate and breast tumors. The table provides a description of these chromosomal regions - length, SCNA type (deletion or amplification), cytoband, beta coefficient, standard error, test statistic, and *p*-value for the AA breast and prostate tumors.

**Additional file 6: Table S5.** Pdf format. Distribution of breast and prostate cancer race-differentiated SCNA defined patient groups by race and breast cancer TNBC subtype status.

**Additional file 7: Table S6.** Pdf format. Differentially expressed genes in the six consistently race-differentiated SCNAs across breast and prostate tumors.

**Additional file 8: Table S7.** Pdf format. Gene-level copy number associations with expression of 18 genes differentially expressed by race in breast and prostate tumors.

**Additional file 9: Figure S1.** Pdf format. Accounting for partially overlapping SCNAs from African American (AA) and European American (EA) races. To account for partially overlapping SCNAs across the two races, we defined new sub-SCNAs based on the overlapping status between the two races, which resulted in sub-SCNAs that are shared by both races or were only identified in one. Three exemplary situations are illustrated, and in each, the actual races are exchangeable. A) In this situation, the SCNA boundaries for each race are distinct from the other, and the boundaries for the AA SCNA are contained within those of the EA SCNA. This results in three distinct SCNAs, with the breakpoints defined by the boundaries of the AA SCNA. B) In this situation, the leftmost boundary is shared by both races, and the rightmost boundary for the AA region is contained within the EA boundary. As a result, two SCNAs are defined based on the rightmost AA boundary. C) In this situation, all of four boundaries are distinct. The first breakpoint is defined by the leftmost AA boundary, and the second breakpoint is defined by the rightmost EA boundary. As a result, three SCNAs are defined.

**Additional file 10: Figure S2.** Pdf format. Calculation of area under the copy number log_2_ ratio curve (cnAUC). An example of a segmented log_2_ ratio curve of an amplification SCNA from one tumor is displayed. Due to the nature of the log_2_ ratio data, the probe specific values fluctuate across the SCNA, with values both above (i.e. gain) and below (i.e. loss) the null reference line of two copies (log_2_ ratio = 0). To quantify the alteration magnitude for this tumor/SCNA, the cnAUC with respect to the null log_2_ ratio value of zero was calculated. Area above reference line (blue region) was treated as positive, and area below the line (orange region) was treated as negative. The sum of positive and negative area is the cnAUC of the region for the tumor.

**Additional file 11: Figure S3.** Pdf format. SCNA burden by race in breast and prostate cancer. Distribution of percentage of the genome altered (ie. SCNA burden) by race in A) breast and B) prostate cancer.

**Additional file 12: Figure S4.** Pdf format. Race permutation results. A) Distribution of the number of SCNA regions with a race-differentiation *p*-value < 0.1 for each permutation in breast tumors. The mean number is 13.2 SCNAs. B) Distribution of number of SCNA regions with a *p*-value < 0.1 for each permutation in prostate tumors. The mean number is 7.2 SCNAs. C) Distribution of the number of overlapping race-differentiated SCNAs with consistent direction of changes in breast and prostate tumors. The average number is 0.56 SCNAs. The blue spikes in A-C indicate the number of observed race-differentiated SCNAs in the current study. D) Length distribution of consistent race-differentiated SCNAs. 10,000 permutations were performed to assess significance.

**Additional file 13: Figure S5.** Pdf format. African American and European American average copy number profiles across chromosome 5q11-q15. A) plots of the breast cancer data and B) plots of the prostate cancer data. In each, the upper panel contains the profile across chromosome 5, and the lower panel highlights the overlapping race-differentiated SCNA region within 5q11-q15 shared by both breast and prostate cancer.

**Additional file 14: Figure S6.** Pdf format. African American and European American average copy number profiles across chromosome 8q21. A) plots of the breast cancer data, and B) plots of the prostate cancer data. In each, the upper panel contains the profile across chromosome 8, and the lower panel highlights the overlapping race-differentiated SCNA region within 8q21 shared by both breast and prostate cancer.

**Additional file 15: Figure S7.** Pdf format. African American and European American average copy number profiles across chromosome 8q21-q24. A) plots of the breast cancer data, and B) plots of the prostate cancer data. In each, the upper panel contains the profile across chromosome 8, and the lower panel highlights the overlapping race-differentiated SCNA region within 8q21-q24 shared by both breast and prostate cancer.

**Additional file 16: Figure S8.** Pdf format. African American and European American average copy number profiles across chromosome 11q22. A) plots of the breast cancer data, and B) plots of the prostate cancer data. In each, the upper panel contains the profile across chromosome 11, and the lower panel highlights the overlapping race-differentiated SCNA region within 11q22 shared by both breast and prostate cancer.

**Additional file 17: Figure S9.**Pdf format. Gap statistic plots for copy number clustering in A) breast and B) prostate cancers. The point at which the gap statistic first reaches a maximum indicates the most likely number of patient groups (clusters) within the cancer types. For both breast and prostate cancer, this occurred at a value of three.

**Additional file 18: Figure S10.** Pdf format. Breast cancer survival for each SCNA-defined breast cancer patient group (BRG) by race. A-C) Kaplan-Meier 10-year overall survival (OS) curves for each BRG, by race. D-F) Kaplan-Meier 10-year progression-free survival (PFS) curves for each BRG, by race.

**Additional file 19: Figure S11.** Pdf format. Prostate cancer progression-free survival (PFS) for each SCNA-defined prostate cancer patient group (PRGs) by race. A-C) Kaplan-Meier 10-year PFS curves for each PRG, by race.

## Data Availability

The data that support the findings of this study are from TCGA and are available in dbGaP, accession number phs000178.v11.p8 (https://ftp.ncbi.nlm.nih.gov/dbgap/studies/phs000178/phs000178.v11.p8/).

## Abbreviations

SCNA: Somatic copy number alterations
AA: African American
EA: European American
TCGA: The Cancer Genome Atlas
PFS: Progression-free survival
ERBB2: Estrogen Growth Factor Receptor 2
PAM50: Prosigna® tumor profiling panel
DNA: Deoxyribonucleic acid
cnAUC: Area under the copy number curve
BRG: Breast group
OS: Overall survival
HR: Hazard Ratio
TNBC: Triple negative breast cancer
PRG: Prostate group
aCGH: array comparative genomic hybridization
ERG: ETS Related gene
PTEN: Phosphatase and tensin homologue
SPINK1: Serine peptidase inhibitor Kazal type 1
SNP: Single nucleoide polymorphism
FDR: False discovery rate

## References

[1] Jemal A, Ward EM, Johnson CJ, Cronin KA, Ma J, Ryerson B, et al. Annual Report to the Nation on the Status of Cancer, 1975-2014, Featuring survival. J Natl Cancer Inst. 2017;109(9):djx030. 10.1093/jnci/djx030 PMC5409140 28376154

[2] Newman LA, Kaljee LM (2017). Health disparities and triple-negative breast Cancer in African American women: a review. JAMA Surg.

[3] Hunter DJ, Riboli E, Haiman CA, Albanes D, Altshuler D, Chanock SJ (2005). A candidate gene approach to searching for low-penetrance breast and prostate cancer genes. Nat Rev Cancer.

[4] Zhao SG, Chang SL, Erho N, Yu M, Lehrer J, Alshalalfa M (2017). Associations of luminal and basal subtyping of prostate Cancer with prognosis and response to androgen deprivation therapy. JAMA Oncol.

[5] Turajlic S, Litchfield K, Xu H, Rosenthal R, McGranahan N, Reading JL (2017). Insertion-and-deletion-derived tumour-specific neoantigens and the immunogenic phenotype: a pan-cancer analysis. Lancet Oncol.

[6] Morris LG, Riaz N, Desrichard A, Senbabaoglu Y, Hakimi AA, Makarov V (2016). Pan-cancer analysis of intratumor heterogeneity as a prognostic determinant of survival. Oncotarget.

[7] Nik-Zainal S, Davies H, Staaf J, Ramakrishna M, Glodzik D, Zou X (2016). Landscape of somatic mutations in 560 breast cancer whole-genome sequences. Nature..

[8] Barbieri CE, Bangma CH, Bjartell A, Catto JW, Culig Z, Gronberg H (2013). The mutational landscape of prostate cancer. Eur Urol.

[9] Vollan HKM, Rueda OM, Chin SF, Curtis C, Turashuili G, Shah S (2015). A tumor DNA complex aberration index is an independent predictor of survival in breast and ovarian cancer. Mol Oncol.

[10] Thompson PA, Brewster AM, Kim-Anh D, Baladandayuthapani V, Broom BM, Edgerton ME (2011). Selective genomic copy number imbalances and probability of recurrence in early-stage breast cancer. PLoS One.

[11] Liu Y, Zhou R, Baumbusch LO, Tsavachidis S, Brewster AM, Do KA (2014). Genomic copy number imbalances associated with bone and non-bone metastasis of early-stage breast cancer. Breast Cancer Res Treat.

[12] Hieronymus H, Schultz N, Gopalan A, Carver BS, Chang MT, Xiao Y (2014). Copy number alteration burden predicts prostate cancer relapse. Proc Natl Acad Sci U S A.

[13] Yu YP, Song C, Tseng G, Ren BG, LaFramboise W, Michalopoulos G (2012). Genome abnormalities precede prostate cancer and predict clinical relapse. Am J Pathol.

[14] Nguyen HG, Welty C, Lindquist K, Ngo V, Gilbert E, Bengtsson H, et al. Validation of GEMCaP as a DNA Based Biomarker to Predict Prostate Cancer Recurrence after Radical Prostatectomy. J Urol. 2017;199(3):719–25. 10.1016/j.juro.2017.09.071 28941923

[15] Levin AM, Lindquist KJ, Avila A, Witte JS, Paris PL, Rybicki BA (2014). Performance of the genomic evaluators of metastatic prostate Cancer (GEMCaP) tumor biomarker for identifying recurrent disease in African American patients. Cancer Epidemiol Biomark Prev.

[16] Rose AE, Satagopan JM, Oddoux C, Zhou Q, Xu R, Olshen AB (2010). Copy number and gene expression differences between African American and Caucasian American prostate cancer. J Transl Med.

[17] Loo LW, Wang Y, Flynn EM, Lund MJ, Bowles EJ, Buist DS (2011). Genome-wide copy number alterations in subtypes of invasive breast cancers in young white and African American women. Breast Cancer Res Treat.

[18] Maples BK, Gravel S, Kenny EE, Bustamante CD (2013). RFMix: a discriminative modeling approach for rapid and robust local-ancestry inference. Am J Hum Genet.

[19] Liu JF, Lichtenberg T, Hoadley KA, Poisson LM, Lazar AJ, Cherniack AD, et al. An Integrated TCGA Pan-Cancer Clinical Data Resource to Drive High-Quality Survival Outcome Analytics. Cell. 2018;173(2):400–16. 10.1016/j.cell.2018.02.052 PMC6066282 29625055

[20] Mermel CH, Schumacher SE, Hill B, Meyerson ML, Beroukhim R, Getz G (2011). GISTIC2.0 facilitates sensitive and confident localization of the targets of focal somatic copy-number alteration in human cancers. Genome Biol.

[21] Tibshirani R, Walther G, Hastie T (2001). Estimating the number of clusters in a data set via the gap statistic. JR Statist Soc B.

[22] Law CW, Chen Y, Shi W, Smyth GK (2014). voom: Precision weights unlock linear model analysis tools for RNA-seq read counts. Genome Biol.

[23] Keenan T, Moy B, Mroz EA, Ross K, Niemierko A, Rocco JW (2015). Comparison of the genomic landscape between primary breast Cancer in African American versus white women and the Association of Racial Differences with Tumor Recurrence. J Clin Oncol.

[24] Ademuyiwa FO, Tao Y, Luo J, Weilbaecher K, Ma CX (2017). Differences in the mutational landscape of triple-negative breast cancer in African Americans and Caucasians. Breast Cancer Res Treat.

[25] Huo D, Hu H, Rhie SK, Gamazon ER, Cherniack AD, Liu J, et al. Comparison of Breast Cancer Molecular Features and Survival by African and European Ancestry in The Cancer Genome Atlas. JAMA Oncol. 2017;3(12):1654–62. 10.1001/jamaoncol.2017.0595 PMC5671371 28472234

[26] Glover TW, Wilson TE, Arlt MF (2017). Fragile sites in cancer: more than meets the eye. Nat Rev Cancer.

[27] Castro P, Creighton CJ, Ozen M, Berel D, Mims MP, Ittmann M (2009). Genomic profiling of prostate cancers from African American men. Neoplasia..

[28] Kumar R, Nagpal G, Kumar V, Usmani SS, Agrawal P, Raghava GPS. HumCFS: a database of fragile sites in human chromosomes. BMC Genomics. 2019;19:985. 10.1186/s12864-018-5330-5 PMC7402404 30999860

[29] DeSantis CE, Fedewa SA, Sauer AG, Kramer JL, Smith RA, Jemal A (2016). Breast Cancer statistics, 2015: convergence of incidence rates between black and white women. CA Cancer J Clin.

[30] Powell IJ, Bock CH, Ruterbusch JJ, Sakr W (2010). Evidence supports a faster growth rate and/or earlier transformation to clinically significant prostate cancer in black than in white American men, and influences racial progression and mortality disparity. J Urol.

[31] Sparano JA, Wang ML, Zhao FM, Stearns V, Martino S, Ligibel JA (2012). Race and hormone receptor-positive breast Cancer outcomes in a randomized chemotherapy trial. Jnci J Natl Cancer I.

[32] Jemal A, Robbins AS, Lin CC, Flanders WD, DeSantis CE, Ward EM (2018). Factors That Contributed to Black-White Disparities in Survival Among Nonelderly Women With Breast Cancer Between 2004 and 2013. J Clin Oncol.

[33] Zoppoli G, Douarre C, Dalla Rosa I, Liu H, Reinhold W, Pommier Y (2011). Coordinated regulation of mitochondrial topoisomerase IB with mitochondrial nuclear encoded genes and MYC. Nucleic Acids Res.

[34] Dick FA, Rubin SM (2013). Molecular mechanisms underlying RB protein function. Nat Rev Mol Cell Biol.

[35] Dyson NJ (2016). RB1: a prototype tumor suppressor and an enigma. Genes Dev.

[36] Witkiewicz AK, Knudsen ES (2014). Retinoblastoma tumor suppressor pathway in breast cancer: prognosis, precision medicine, and therapeutic interventions. Breast Cancer Res.

[37] Kubota Y, Fujinami K, Uemura H, Dobashi Y, Miyamoto H, Iwasaki Y (1995). Retinoblastoma gene mutations in primary human prostate cancer. Prostate..

[38] Sharma A, Yeow WS, Ertel A, Coleman I, Clegg N, Thangavel C (2010). The retinoblastoma tumor suppressor controls androgen signaling and human prostate cancer progression. J Clin Invest.

[39] Tseng YY, Moriarity BS, Gong WM, Akiyama R, Tiwari A, Kawakami H, et al. PVT1 dependence in cancer with MYC copy-number increase. Nature. 2014;512(7512):82–6. 10.1038/nature13311 PMC4767149 25043044

