# Supplementary material for: Breast and prostate cancers harbor common somatic copy number alterations that consistently differ by race and are associated with survival
Source: BMC Med Genomics. 2020 Aug 20;13:116. doi: 10.1186/s12920-020-00765-2 (PMC7441621; doi:10.1186/s12920-020-00765-2)
Supplement: Supplementary file 1 — Additional file 1. Supplementary Methods. Pdf format. The document describes the following methods used in the analysis: estimating local ancestry and validating self-reported race; identifying recurrent SCNAs through Gistic2; integration of SCNA regions identified by Gistic2 in African American (AA) and European American (EA) tumors separately; calculation of SCNA magnitude for each tumor using an area under the copy number curve approach; assessment for multiple testing using a permutation approach; and association of local ancestry with race-differentiated SCNAs. [file 12920_2020_765_MOESM1_ESM.pdf]

Supplementary Methods

Estimating local ancestry and validating self-reported Race

Regional chromosomal ancestry for all subjects in this study was inferred using RFMix (43), with the YRI (Yoruba in Ibadan, Nigeria) and CEU (Utah residents with northern and western ancestry) subpopulations from the 1000 Genome Project (44) used as the reference panels for the respective African and European ancestral populations contributing to AA genomes. After inference of regional chromosomal ancestry, we validated the self-reported race of subjects by comparing it with their corresponding genome-wide ancestry proportion, which was calculated for each subject by dividing the number of African alleles over total number of alleles across the autosomes.

Identifying recurrent SCNAs through Gistic2

To filter out background variation in copy number, amplification and deletion thresholds of the $log_2(copy\ number/2)$ quantity were set at 0.3 and -0.3 respectively. Gistic2 uses base pair length to separate focal and arm-level SCNAs, and this arm-level threshold was set at 0.95. The Benjamini-Hochberg false discovery rate (FDR) procedure (47) was used in Gistic2 to correct for multiple testing, and a FDR of 0.35 was set as the significance threshold in the analysis, which is the suggested default threshold for the program.

Integration of SCNA regions identified by Gistic2 in African American (AA) and European American (EA) tumors separately

With larger samples sizes for the EA relative to AA subjects in both breast and prostate cancer datasets, SCNAs were identified using Gistic2 in each tumor type separately by race in

order to ensure that race-specific SCNAs were not missed. In order to test for SCNA differences between EA and AA tumors, differences between the EA and AA SCNAs needed to be reconciled. While Gistic2 defined SCNA boundaries without overlap between the races posed no discrepancy, SCNA boundaries needed to be established for those that had some degree of overlap between the two races (Figure S1). Considering that portions of an SCNA identified in only one race are more likely to have significantly different copy number changes in comparison to the other, a true copy number difference could be averaged-out if we were to simply take the union of two overlapping SCNAs (i.e. if the SCNA boundaries are established based on the extremes of the boundaries from either race) to make a single region. To avoid that potential dilution effect, we divided the non-overlapping regions into multiple smaller sub-regions. Figure S1 shows the three ways that overlapping Gistic2 defined regions from the two races could partially overlap, and how the new SCNAs were defined. This approach resulted in sub-SCNAs that were shared between the two races and those that were racially distinct.

Calculation of SCNA magnitude for each tumor using an area under the copy number curve approach

It is a challenge to measure the copy number over a region due to the fluctuation of $\log_2$ ratios called from array data. This fluctuation exists even after copy number segmentation. While categorizing the SCNA strictly based on amplification/deletion status is one possible solution, this approach ignores the magnitude information contained within the individual probes, which may contain meaningful degree of alteration data. To capture both information of alteration state (i.e. amplification/deletion status) as well as the magnitude of copy number change, we used the area under the copy number $\log_2$ ratio curve (cnAUC) with respect to the null value of two copies (i.e.

log_2 ratio=0). An example of a log_2 ratio profile for a tumor across an SCNA and the calculation of the corresponding cnAUC value is provided in Figure S2. Regions above the null reference line represent positive area (amplification), and those below the reference line represent negative area (deletion). For each tumor, the sum of positive and negative area is the cnAUC of the SCNA for the tumor. A region with no copy number changes will have an cnAUC close to zero, and a cnAUC significantly above or below zero suggests an amplification or deletion SCNA, respectively.

Assessment for multiple testing using a permutation approach

Given the correlation between adjacent SCNA regions in the two datasets as well as the a more liberal threshold of statistical significance of race-differentiated SCNAs ($p<0.1$), it is important to determine whether the total race-differentiated SCNAs identified by tumor type as well as the observed six race-differentiated SCNAs common to both tumor types were more than what would be expected by chance alone. Specifically, the race identifier was permuted to generate a null distribution of the total number of race-differentiated SCNAs observed by chance alone. The race of each tumor was randomly selected while keeping the overall AA/EA ratio the same as observed in the TCGA data, and linear modeling as described above was performed to assess the association between cnAUC and race. The same threshold of 0.1 was used to identify race-differentiated SCNAs from linear regression in single tumor type. Based on 10,000 permutations, the expected number of race-differentiated SCNAs in breast cancer is 13.2 and in prostate cancer is 7.2 (Figure S8, A and B). The probability of observing 58 race-differentiated SCNAs in breast at significance level of 0.1 is less than 10^{-4}(i.e. a value not observed in any of the 10,000 permutations), and the probability of observing 21 race-differentiated SCNAs in prostate at significance level of 0.1 is 0.0064. The expected number of consistent race-differentiated

SCNAs common to both tumor types is 0.56 with average length of 4 Mbp while the probability of observing six or more consistent race-differentiated SCNAs is 0.0007.

Association of local ancestry with race-differentiated SCNAs

To quantify the relationship between SCNAs and regional ancestry- calculated as percentage of African alleles across each SCNA in AA tumors, the following linear model was used:

$$cnAUC = regional\ ancestry + age + tumor\ pathology + \ genome\ wide\ ancestry$$

In addition to age at diagnosis and tumor pathology, this model also included adjustment for genome-wide African ancestry.
