# Supplementary material for: Breast and prostate cancers harbor common somatic copy number alterations that consistently differ by race and are associated with survival
Source: BMC Med Genomics. 2020 Aug 20;13:116. doi: 10.1186/s12920-020-00765-2 (PMC7441621; doi:10.1186/s12920-020-00765-2)
Supplement: Supplementary file 2 — Additional file 2: Table S1. Pdf format. Somatic copy number alterations (SCNAs) in TCGA breast tumors (n = 886) that differ significantly by race. 58 SCNAs that showed significant differences by race in breast tumors are listed in this table along with information on their chromosomal location, length, SCNA type (deletion or amplification), cytoband, beta coefficient, standard error, test statistic, and p-value. [file 12920_2020_765_MOESM2_ESM.pdf]

Table S1. Somatic copy number alterations (SCNAs) in TCGA breast tumors (n=886) that differ significantly by race.

Chromosome number: base pair position start-end*	Length in megabases	SCNA type	Cytoband	Beta Coefficient†	SE	T-statistic	P-value
3:154227886-169450068	15.22	Amp	q25.2-q26.2	0.026	0.016	1.61	0.107
3:169450069-171879450	2.43	Amp	q26.2-q26.31	0.035	0.019	1.91	0.057
4:109489756-109493251	0.00	Del	q25	-0.017	0.009	-1.79	0.074
4:176731730-182822332	6.09	Del	q34.2-q34.3	-0.034	0.018	-1.86	0.064
4:182822333-191154275	8.33	Del	q34.3-q35.2	-0.031	0.013	-2.37	0.018
4:3369932-3431340	0.06	Del	p16.3	-0.032	0.017	-1.89	0.059
5:160562330-165741478	5.18	Del	q34	-0.070	0.019	-3.68	0.000
5:51204966-51924477	0.72	Del	q11.2	-0.055	0.020	-2.76	0.006
5:51924478-88003401	36.08	Del	q11.2-q14.3	-0.083	0.019	-4.34	0.000
5:88003402-114971116	26.97	Del	q14.3-q22.3	-0.087	0.019	-4.67	0.000
6:105142579-105544119	0.40	Amp	q16.3-q21	0.066	0.028	2.34	0.020
6:105544120-107830797	2.29	Amp	q21	0.074	0.031	2.42	0.016
6:107830798-108690475	0.86	Amp	q21	0.061	0.030	2.00	0.046
6:152481982-170769426	18.29	Del	q25.1-q27	0.038	0.022	1.79	0.075
6:170769427-171115066	0.35	Del	q27	0.019	0.009	2.18	0.029
6:21312556:22614340	1.30	Amp	p22.3	0.035	0.021	1.70	0.089
6:2232105-4756175	2.52	Del	p25.3-p25.1	0.050	0.021	2.41	0.016
6:22614341-22817848	0.20	Amp	p22.3	0.036	0.020	1.75	0.080
6:6754109-21312555	14.56	Amp	p25.1-p22.3	0.057	0.019	3.00	0.003
6:69393208-83933646	14.54	Del	q12-q14.2	0.036	0.020	1.86	0.063
6:99929476:152481981	52.55	Del	q16.2-q25.1	0.037	0.021	1.74	0.082
7:151140793-151976410	0.84	Del	q36.1	0.035	0.021	1.71	0.088
7:151981957:153770265	1.79	Del	q36.1-q36.2	0.035	0.020	1.76	0.079
7:32999602-33047676	0.05	Amp	p14.3	-0.034	0.015	-2.28	0.023
8:1-37953889	37.95	Del	p23.3-p11.23	-0.045	0.027	-1.67	0.095
8:61177663-146364021	85.19	Amp	q12.1-q24.3	0.070	0.028	2.51	0.012
9:1-18127677	18.13	Del	p24.3-p22.2	0.035	0.021	1.70	0.090

Table S1. Somatic copy number alterations (SCNAs) in TCGA breast tumors (n=886) that differ significantly by race.

Chromosome number: base pair position start-end*	Length in megabases	SCNA type	Cytoband	Beta Coefficient†	SE	T-statistic	P-value
9:22147714-30113862	7.97	Del	p21.3-p21.1	0.057	0.024	2.45	0.015
10:1-3730104	3.73	Amp	p15.3-p15.2	0.072	0.018	3.99	0.000
10:3730105-6398980	2.67	Amp	p15.2-p15.1	0.081	0.021	3.91	0.000
10:61852107-62575303	0.72	Amp	q21.2	0.053	0.021	2.57	0.011
10:6398981-14643449	8.24	Amp	p15.1-p13	0.086	0.020	4.30	0.000
11:1-766166	0.77	Del	p15.5	-0.021	0.008	-2.64	0.008
11:64592727-65674138	1.08	Del	q13.1	-0.038	0.018	-2.14	0.033
11:65766045-66641072	0.88	Amp	q13.1-q13.2	-0.053	0.022	-2.46	0.014
11:66641073-68027183	1.39	Amp	q13.2	-0.048	0.025	-1.93	0.054
11:68027184-68168228	0.14	Amp	q13.2	-0.075	0.027	-2.77	0.006
11:74235492-135006515	60.77	Del	q13.4-q25	-0.037	0.019	-1.94	0.052
11:766167-11684113	10.92	Del	p15.5-p15.3	-0.047	0.018	-2.65	0.008
12:11703865-12303099	0.60	Amp	p13.2	0.040	0.025	1.62	0.105
12:1-2306551	2.31	Amp	p13.33	0.024	0.013	1.78	0.076
13:30777606-30978054	0.20	Amp	q12.3	-0.050	0.024	-2.11	0.035
13:31949050-38243466	6.29	Del	q12.3-q13.3	-0.044	0.022	-2.03	0.042
13:38243467-52321574	14.08	Del	q13.3-q14.3	-0.051	0.022	-2.34	0.020
13:52321575-59628349	7.31	Del	q14.3-q21.2	-0.048	0.022	-2.23	0.026
14:66905865-67198116	0.29	Del	q23.3	-0.054	0.018	-2.93	0.004
14:67198117-70999022	3.80	Del	q23.3-q24.2	-0.054	0.019	-2.88	0.004
14:70999023-97705236	26.71	Del	q24.2-q32.2	-0.036	0.018	-2.02	0.043
15:1-37513451	37.51	Del	p13-q14	-0.013	0.007	-1.88	0.060
15:37513452-43466475	5.95	Del	q14-q15.2	-0.046	0.018	-2.53	0.012
16:55240635-58196161	2.96	Del	q12.2-q21	0.046	0.025	1.88	0.060
16:58196162-90354752	32.16	Del	q21-q24.3	0.066	0.023	2.83	0.005
17:45603013-45969605	0.37	Amp	q21.32	-0.039	0.023	-1.73	0.084
19:1-4677580	4.68	Del	p13.3	-0.035	0.015	-2.37	0.018

Table S1. Somatic copy number alterations (SCNAs) in TCGA breast tumors (n=886) that differ significantly by race.

Chromosome number: base pair position start-end*	Length in megabases	SCNA type	Cytoband	Beta Coefficient†	SE	T-statistic	P-value
19:29282210-29315606	0.03	Amp	q12	0.046	0.022	2.12	0.035
19:29315607-31229637	1.91	Amp	q12	0.076	0.022	3.49	0.001
19:31229638-36069787	4.84	Amp	q12-q13.12	0.053	0.016	3.20	0.001
19:55573038-58776089	3.20	Amp	q13.42-q13.43	-0.032	0.018	-1.75	0.081

Abbreviations: SCNA=somatic copy number alteration; Del=deletion; Amp=amplification; AA=African American; EA=European American; SE=standard error of the beta coefficient.

*Based on human genome build hg19

† Beta coefficient for the "race" term in the linear model "cnAUC=race+age+tumor pathology"

In amplified regions, a positive beta coefficient signifies a greater degree of amplification among AAs relative to EAs.

In deletion regions, a negative beta coefficient signifies a greater degree of DNA loss among AAs relative to EAs.
