# Supplementary material for: Breast and prostate cancers harbor common somatic copy number alterations that consistently differ by race and are associated with survival
Source: BMC Med Genomics. 2020 Aug 20;13:116. doi: 10.1186/s12920-020-00765-2 (PMC7441621; doi:10.1186/s12920-020-00765-2)
Supplement: Supplementary file 3 — Additional file 3: Table S2. Pdf format. SCNAs in TCGA prostate tumors (n = 309) that differ significantly by race. 21 SCNAs that showed significant differences by race in prostate tumors are listed in this table along with information on their chromosomal location, length, SCNA type (deletion or amplification), cytoband, beta coefficient, standard error, test statistic, and p-value. [file 12920_2020_765_MOESM3_ESM.pdf]

Table S2. SCNAs in TCGA prostate tumors (n=309) that differ significantly by race.

Chromosome number: base pair position start-end*	Length in megabases	SCNA type	Cytoband	Beta Coefficient†	SE	T-statistic	P-value
1:95625034-95638642	0.01	Del	p21.3	-0.011	0.007	-1.65	0.100
1:192415363-192664591	0.25	Del	q31.2	-0.080	0.021	-3.80	0.000
2:117472243-119706070	2.23	Del	q14.1-q14.2	-0.074	0.027	-2.76	0.006
2:119706071-142334434	22.63	Del	q14.2-q22.2	-0.099	0.033	-2.99	0.003
2:146503972-152676739	6.17	Del	q22.3-q23.3	-0.050	0.030	-1.65	0.100
5:50124857-80506380	30.38	Del	q11.1-q14.1	-0.062	0.033	-1.87	0.062
5:94139355-102688824	8.55	Del	q15-q21.1	-0.116	0.044	-2.61	0.009
6:67715998-68704380	0.99	Del	q12	-0.088	0.028	-3.11	0.002
6:68704381-123268214	54.56	Del	q12-q22.31	-0.086	0.032	-2.70	0.007
7:126235976-127148840	0.91	Del	q31.33-q32.1	0.086	0.031	2.76	0.006
8:78097650-84211555	6.11	Amp	q21.11-q21.13	0.088	0.039	2.24	0.026
8:90059223-144506437	54.45	Amp	q21.3-q24.3	0.087	0.036	2.44	0.015
8:144506438-144568457	0.06	Amp	q24.3	0.046	0.025	1.86	0.064
10:57386964-58273391	0.89	Del	q21.1	-0.173	0.045	-3.83	0.000
11:108993953-109543386	0.55	Del	q22.3	-0.071	0.027	-2.61	0.010
13:31720389-84976297	53.26	Del	q12.3-q31.1	-0.069	0.034	-2.02	0.045
15:25436069-25453012	0.02	Amp	q11.2	-0.040	0.021	-1.86	0.064
16:46640973-47393622	0.75	Amp	q11.2-q12.1	0.066	0.033	2.01	0.046
16:60711210-90354752	29.64	Del	q21-q24.3	-0.081	0.036	-2.25	0.025
21:39850021-42832638	2.98	Del	q22.2-q22.3	0.099	0.047	2.11	0.036
21:42832639-42868335	0.04	Del	q22.3	0.097	0.046	2.10	0.036

Abbreviations: SCNA=somatic copy number alteration; Del=deletion; Amp=amplification; AA=African American; EA=European American; SE=standard error of the beta coefficient.

*Based on human genome build hg19

† Beta coefficient for the "race" term in the linear model "cnAUC=race+age+tumor pathology"

In amplified regions, a positive beta coefficient signifies a greater degree of amplification among AAs relative to EAs.

In deletion regions, a negative beta coefficient signifies a greater degree of DNA loss among AAs relative to EAs.
