# Supplementary material for: Breast and prostate cancers harbor common somatic copy number alterations that consistently differ by race and are associated with survival
Source: BMC Med Genomics. 2020 Aug 20;13:116. doi: 10.1186/s12920-020-00765-2 (PMC7441621; doi:10.1186/s12920-020-00765-2)
Supplement: Supplementary file 4 — Additional file 4 Table S3. Pdf format. Additional cross-tumor validation of the six race-differentiated SCNAs in TCGA. [file 12920_2020_765_MOESM4_ESM.pdf]

Table S3. Additional cross-tumor* validation of the six race-differentiated SCNAs in TCGA.

Breast and Prostate cancer SCNA discovery			Other cancer type SCNA validation							
Cytoband†	Chromosome position†	Breast and prostate SCNA type	Cancer Type*	Chromosome position†	Length (Mb)	SCNA type	Beta coefficient†	SE	T-statistic	P-value
5q11.2-q14.1	5:51204966-80506380	Del	Endometrial	5:50124857-63329958	13.21	Del	-0.023	0.022	-1.03	0.301
				5:63329959-94282979	30.95	Del	-0.016	0.021	-0.80	0.426
			Ovarian	5:45378457-55750914	10.37	Del	0.088	0.037	2.37	0.018
				5:55750915-59044767	3.29	Del	0.185	0.082	2.27	0.024
				5:59044768-62065114	3.02	Del	0.175	0.071	2.48	0.014
				5:62065115-125175058	63.11	Del	0.064	0.056	1.13	0.258
			Lung	5:52284387-58615497	6.33	Del	-0.047	0.038	-1.22	0.224
				5:58615498-58707776	0.09	Del	-0.042	0.037	-1.13	0.260
				5:58707777-64444922	5.74	Del	-0.019	0.036	-0.52	0.600
				5:64444923-136407584	71.96	Del	-0.016	0.034	-0.47	0.641
			Kidney	NA	NA	NA	NA	NA	NA	NA
5q15-q21.1	5:94139355-102688824	Del	Endometrial	5:63329959-94282979	30.95	Del	-0.016	0.021	-0.80	0.426
			Ovarian	5:62065115-125175058	63.11	Del	0.064	0.056	1.13	0.258
			Lung	5:64444923-136407584	71.96	Del	-0.016	0.034	-0.47	0.641
			Kidney	5:88862287-103693025	14.83	Amp	-0.026	0.032	-0.80	0.425
8q21.11-q21.	8:78097650-84211555	Amp	Endometrial	NA	NA	NA	NA	NA	NA	NA
			Ovarian	8:65946564-115168901	49.22	Amp	-0.144	0.061	-2.38	0.018
			Lung	8:64945035-93539198	28.59	Amp	-0.027	0.041	-0.66	0.507
			Kidney	NA	NA	NA	NA	NA	NA	NA
8q21.3-q24.3	8:90059223-144568457	Amp	Endometrial	8:95579787-99567648	3.99	Amp	0.045	0.030	1.50	0.135
				**8:100144058-100907359[§]**	**0.76**	**Amp**	**0.055**	**0.030**	**1.87**	**0.063**
				**8:100907360-140610028[§]**	**39.70**	**Amp**	**0.070**	**0.028**	**2.49**	**0.013**
				**8:140610029-143946427[§]**	**3.34**	**Amp**	**0.052**	**0.030**	**1.74**	**0.082**
			Ovarian	8:65946564-115168901	49.22	Amp	-0.144	0.061	-2.38	0.018
				8:115168902-146364021	31.20	Amp	-0.076	0.077	-0.98	0.326
			Lung	8:93539199-143507187	49.97	Amp	-0.045	0.040	-1.12	0.262
				8:143507188-146364021	2.86	Amp	-0.048	0.027	-1.80	0.073
			Kidney	8:130667622-130732818	0.07	Amp	-0.001	0.027	-0.02	0.983
11q22.3	11:108993953-109543386	Del	Endometrial	11:83791774-120931054	37.14	Del	-0.025	0.019	-1.32	0.189
			Ovarian	NA	NA	NA	NA	NA	NA	NA
			Lung	11:104239997-113587551	9.35	Del	-0.012	0.031	-0.40	0.692
			Kidney	NA	NA	NA	NA	NA	NA	NA

13q12.3-q21.	13:31949050-59628349	Del	Endometrial	**13:31392655-46090461§**	**14.70**	**Del**	**-0.039**	**0.023**	**-1.68**	**0.094**
				13:46090462-50019374	3.93	Del	-0.028	0.025	-1.12	0.261
				13:50019375-61187197	11.17	Del	-0.031	0.024	-1.31	0.192
			Ovarian	**13:31158332-40490760§**	**9.33**	**Del**	**-0.169**	**0.067**	**-2.52**	**0.012**
				13:40490761-40716260	0.23	Del	-0.113	0.071	-1.59	0.113
				**13:40716261-51631042§**	**10.91**	**Del**	**-0.138**	**0.068**	**-2.03**	**0.043**
				13:51631043-56802296	5.17	Del	-0.097	0.070	-1.39	0.164
			Lung	13:19650694-85435149	65.78	Del	0.020	0.031	0.64	0.522
			Kidney	NA	NA	NA	NA	NA	NA	NA

Abbreviations - AA=African American; Amp=Amplification; Del=Deletion; EA=European American; NA = no recurrent SCNAs were identified by Gistic2; SE=Standard error of the coefficient.

* The following four tumor types were evaluated along with their sAmple sizes - Endometrial (N: AA=102, EA=360); Ovarian (N: AA=31, EA=453); Lung (N: AA=80, EA=735); Kidney (N: AA=118, EA=711).

† positive beta coefficient signifies greater DNA Amplification in AA relative to EA.
negative beta coefficient signifies greater DNA Deletion in AA relative to EA.

‡ Based on human genome build hg19

§ SCNAs where significant race difference coefficients were consistent in direction with the breast and prostate cancer results (i.e, more extreme gain and loss in AA compared to EA).
