# Supplementary material for: Breast and prostate cancers harbor common somatic copy number alterations that consistently differ by race and are associated with survival
Source: BMC Med Genomics. 2020 Aug 20;13:116. doi: 10.1186/s12920-020-00765-2 (PMC7441621; doi:10.1186/s12920-020-00765-2)
Supplement: Supplementary file 5 — Additional file 5: Table S4. Pdf format. Association of regional chromosomal African ancestry with SCNAs among African Americans in the six regions where African Americans had higher magnitude SCNAs relative to European Americans in both prostate and breast tumors. The table provides a description of these chromosomal regions - length, SCNA type (deletion or amplification), cytoband, beta coefficient, standard error, test statistic, and p-value for the AA breast and prostate tumors. [file 12920_2020_765_MOESM5_ESM.pdf]

Table S4. Association of regional chromosomal African ancestry with SCNAs among African Americans in the six regions where African Americans had higher magnitude SCNAs relative to European Americans in both prostate and breast tumors.

				AA Breast TCGA Tumors				AA Prostate TCGA Tumors			
**Chromosome number: base pair position start-end***	**Length (Mb)**	**SCNA type**	**Cytoband**	**Beta Coefficient†**	**SE**	**T-statistic**	**P-value**	**Beta Coefficient†**	**SE**	**T-statistic**	**P-value**
5:51204966-80506380	29.3	Del	q11.2-q14.1	0.111	0.098	1.13	0.259	0.260	0.227	1.14	0.260
5:94139355-102688824	8.55	Del	q15-q21.1	0.076	0.067	1.12	0.264	-0.065	0.363	-0.18	0.858
8:78097650-84211555	6.11	Amp	q21.11-q21.13	0.013	0.120	0.11	0.911	-0.003	0.167	-0.02	0.986
8:90059223-144568457	54.51	Amp	q21.3-q24.3	0.061	0.183	0.34	0.737	0.438	0.364	1.20	0.237
11:108993953-109543386	0.55	Del	q22.3	0.004	0.087	0.05	0.960	0.097	0.158	0.62	0.542
13:31949050-59628349	27.68	Del	q12.3-q21.3	-0.011	0.111	-0.10	0.924	-0.016	0.156	-0.10	0.921

Abbreviations: SCNA=somatic copy number alteration; Del=deletion; Amp=amplification; AA=African American; SE=standard error of the beta coefficient.

*Based on human genome build hg19

† Beta coefficient for the "regional ancestry" term in the linear model "cnAUC=regional ancestry+age+tumor pathology+ genome wide ancestry "

In amplified regions, a positive beta coefficient indicates that higher degree of amplification is associated with increasing African ancestry within the SCNA.

In deletion regions, a negative beta coefficient indicates that a higher degree of amplification is associated with increasing African ancestry within the SCNA.
