# Supplementary material for: Breast and prostate cancers harbor common somatic copy number alterations that consistently differ by race and are associated with survival
Source: BMC Med Genomics. 2020 Aug 20;13:116. doi: 10.1186/s12920-020-00765-2 (PMC7441621; doi:10.1186/s12920-020-00765-2)
Supplement: Supplementary file 6 — Additional file 6: Table S5. Pdf format. Distribution of breast and prostate cancer race-differentiated SCNA defined patient groups by race and breast cancer TNBC subtype status. [file 12920_2020_765_MOESM6_ESM.pdf]

Table S5: Distribution of breast and prostate cancer race-differentiated SCNA defined patient groups by race and breast cancer TNBC subtype status.

		Race			Subtype		
Cancer type	SCNA Group	AA	EA	P-value*	TNBC	non-TNBC	P-value*
Breast	BRG1	105(64%)	519(75.5%)	0.01	88(61.5%)	500(75.6%)	0.003
	BRG2	6(3.7%)	16(2.3%)		5(3.5%)	16(2.4%)	
	BRG3	53(32.3%)	152(22.1%)		50(35.0%)	145(21.9%)	
Prostate	PRG1	32(76.2%)	227(84.1%)	0.069	-	-	-
	PRG2	5(11.9%)	10(3.7%)		-	-	
	PRG3	5(11.9%)	33(12.2%)		-	-	

Abbreviations: SCNA=somatic copy number alteration; AA=African American; EA=European American; BRG1-3=three molecular breast cancer patient groups based on race-differentiated SCNAs shared with prostate cancer; PRG1-3=three molecular prostate cancer patient groups based on race-differentiated SCNAs shared with breast cancer; TNBC=triple negative breast cancer.
*P-value from a chi-square test.
