# Supplementary material for: Breast and prostate cancers harbor common somatic copy number alterations that consistently differ by race and are associated with survival
Source: BMC Med Genomics. 2020 Aug 20;13:116. doi: 10.1186/s12920-020-00765-2 (PMC7441621; doi:10.1186/s12920-020-00765-2)
Supplement: Supplementary file 7 — Additional file 7: Table S6. Pdf format. Differentially expressed genes in the six consistently race-differentiated SCNAs across breast and prostate tumors. [file 12920_2020_765_MOESM7_ESM.pdf]

Table S6. Differentially expressed genes in the six consistently race-differentiated SCNAs across breast and prostate tumors

		Breast Tumors				Prostate Tumors			
**Gene Symbol**	**Cytoband**	**Log_2 fold change***	**T-statistic**	**P value**	**FDR†**	**Log_2 fold change***	**T-statistic**	**P value**	**FDR†**
TOP1MT	8q24.3	0.87	12.42	7.65E-33	3.99E-30	0.62	5.96	7.21E-09	3.76E-06
GLI4	8q24.3	1.10	12.00	6.32E-31	1.65E-28	0.47	3.83	1.55E-04	1.01E-02
RB1	13q14.2	-0.75	-10.55	1.29E-24	5.62E-23	-0.27	-3.34	9.28E-04	2.55E-02
GUSBP3	5q13	-1.20	-10.15	5.04E-23	1.38E-21	-0.85	-5.42	1.24E-07	2.15E-05
KBTBD7	13q14.11	-0.88	-9.58	9.05E-21	1.63E-19	-0.44	-3.47	5.94E-04	2.02E-02
MARVELD2	5q13.2	-0.63	-8.02	3.19E-15	2.38E-14	-0.50	-4.85	1.99E-06	2.60E-04
KBTBD6	13q14.11	-0.37	-7.24	9.55E-13	5.33E-12	-0.37	-3.85	1.47E-04	1.01E-02
OCLN	5q13.2	-0.69	-7.06	3.25E-12	1.73E-11	-0.50	-3.25	1.27E-03	3.31E-02
ARSB	5q14.1	-0.40	-6.43	2.06E-10	8.76E-10	-0.33	-3.58	3.98E-04	1.73E-02
WISP1	8q24.22	-0.72	-5.79	9.46E-09	3.03E-08	0.61	3.65	3.08E-04	1.61E-02
PVT1	8q24.21	0.41	4.83	1.58E-06	4.14E-06	0.44	3.35	9.25E-04	2.55E-02
SOHLH2	13q13.3	-0.79	-4.82	1.64E-06	4.27E-06	-1.48	-3.16	1.74E-03	3.87E-02
MTERFD1/MTERF3	8q22.1	0.22	3.59	3.52E-04	6.38E-04	0.26	3.48	5.78E-04	2.02E-02
C11orf87	11q22.3	-0.44	-3.39	7.29E-04	1.28E-03	0.87	3.10	2.11E-03	4.08E-02
CDADC1	13q14.2	-0.13	-3.13	1.81E-03	3.07E-03	-0.23	-3.04	2.59E-03	4.83E-02
SNX31	8q22.3	0.43	2.70	7.05E-03	1.10E-02	1.69	5.63	4.22E-08	1.10E-05
SUGT1	13q14.3	0.10	2.44	1.47E-02	2.14E-02	-0.21	-3.13	1.93E-03	4.04E-02
PELO	5q11.2	-0.08	-2.30	2.19E-02	3.10E-02	-0.23	-3.85	1.47E-04	1.01E-02

Abbreviations: AA=African American; EA=European American; FDR=False Discovery Rate

* log2.Fold.Change is the log2(expression in AA Tumors/expression in EA Tumors).

† Adjusted pvalue using Benjamini-Hochberg False Discovery Rate method.
