# Supplementary material for: Breast and prostate cancers harbor common somatic copy number alterations that consistently differ by race and are associated with survival
Source: BMC Med Genomics. 2020 Aug 20;13:116. doi: 10.1186/s12920-020-00765-2 (PMC7441621; doi:10.1186/s12920-020-00765-2)
Supplement: Supplementary file 8 — Additional file 8: Table S7. Pdf format. Gene-level copy number associations with expression of 18 genes differentially expressed by race in breast and prostate tumors. [file 12920_2020_765_MOESM8_ESM.pdf]

Table S7. Gene-level copy number associations with expression of 18 genes differentially expressed by race in breast and prostate tumors

		Breast Tumors				Prostate Tumors			
**Gene Symbol**	**Cytoband**	**Beta coefficient***	**T-statistic**	**P value**	**FDR†**	**Beta coefficient***	**T-statistic**	**P value**	**FDR†**
TOP1MT	8q24.3	0.53	15.16	2.65E-46	**7.94E-46**	0.44	7.55	5.33E-13	**1.07E-12**
GLI4	8q24.3	0.51	10.63	6.63E-25	**1.08E-24**	0.48	7.29	2.75E-12	**4.96E-12**
RB1	13q14.2	0.67	14.75	3.53E-44	**9.06E-44**	0.29	9.38	1.70E-18	**5.09E-18**
GUSBP3	5q13	1.12	14.04	1.40E-40	**3.15E-40**	0.41	4.32	2.12E-05	**2.94E-05**
KBTBD7	13q14.11	0.56	8.95	2.14E-18	**3.21E-18**	0.32	6.13	2.82E-09	**4.24E-09**
MARVELD2	5q13.2	0.69	13.42	1.70E-37	**3.40E-37**	0.45	7.78	1.15E-13	**2.59E-13**
KBTBD6	13q14.11	0.49	15.82	8.21E-50	**3.69E-49**	0.36	10.06	1.08E-20	**4.84E-20**
OCLN	5q13.2	0.50	7.29	6.83E-13	**9.46E-13**	0.37	4.09	5.58E-05	**6.69E-05**
ARSB	5q14.1	-0.01	-0.16	0.869	0.869	0.38	6.23	1.60E-09	**2.61E-09**
WISP1	8q24.22	-0.01	-0.22	0.824	0.869	-0.07	-0.70	0.485	0.494
PVT1	8q24.21	0.61	15.63	7.98E-49	**2.87E-48**	0.56	7.83	8.65E-14	**2.22E-13**
SOHLH2	13q13.3	0.46	4.29	2.01E-05	**2.58E-05**	0.96	4.11	5.06E-05	**6.50E-05**
MTERFD1/MTERF3	8q22.1	0.67	26.12	6.50E-112	**1.17E-110**	0.40	10.64	1.24E-22	**7.41E-22**
C11orf87	11q22.3	0.10	1.26	0.209	0.235	-0.15	-0.68	0.494	0.494
CDADC1	13q14.2	0.43	17.42	1.19E-58	**7.15E-58**	0.34	13.58	4.60E-33	**4.14E-32**
SNX31	8q22.3	0.18	2.10	0.036	**0.043**	0.44	2.34	0.020	**0.023**
SUGT1	13q14.3	0.45	18.98	1.12E-67	**1.01E-66**	0.34	15.37	1.04E-39	**1.87E-38**
PELO	5q11.2	0.30	13.29	7.05E-37	**1.27E-36**	0.36	9.97	2.08E-20	**7.49E-20**

Abbreviations: AA=African American; EA=European American; FDR=False Discovery Rate

* log2.Fold.Change is the log2(expression in AA Tumors/expression in EA Tumors).

† Adjusted pvalue using Benjamini-Hochberg False Discovery Rate method.
