# Supplementary figures and images for: Breast and prostate cancers harbor common somatic copy number alterations that consistently differ by race and are associated with survival
Source: BMC Med Genomics. 2020 Aug 20;13:116. doi: 10.1186/s12920-020-00765-2 (PMC7441621; doi:10.1186/s12920-020-00765-2)

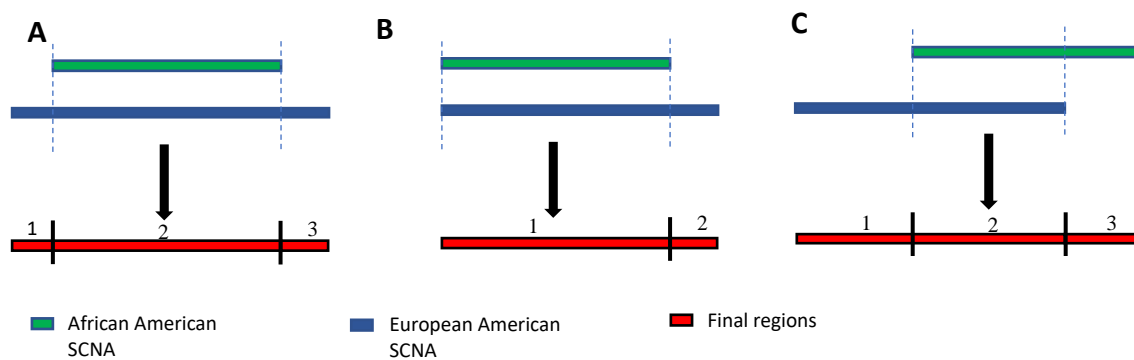

**Figure S1**

Supplement: Supplementary file 9 — Additional file 9: Figure S1. Pdf format. Accounting for partially overlapping SCNAs from African American (AA) and European American (EA) races. To account for partially overlapping SCNAs across the two races, we defined new sub-SCNAs based on the overlapping status between the two races, which resulted in sub-SCNAs that are shared by both races or were only identified in one. Three exemplary situations are illustrated, and in each, the actual races are exchangeable. A) In this situation, the SCNA boundaries for each race are distinct from the other, and the boundaries for the AA SCNA are contained within those of the EA SCNA. This results in three distinct SCNAs, with the breakpoints defined by the boundaries of the AA SCNA. B) In this situation, the leftmost boundary is shared by both races, and the rightmost boundary for the AA region is contained within the EA boundary. As a result, two SCNAs are defined based on the rightmost AA boundary. C) In this situation, all of four boundaries are distinct. The first breakpoint is defined by the leftmost AA boundary, and the second breakpoint is defined by the rightmost EA boundary. As a result, three SCNAs are defined. [file 12920_2020_765_MOESM9_ESM.pdf]

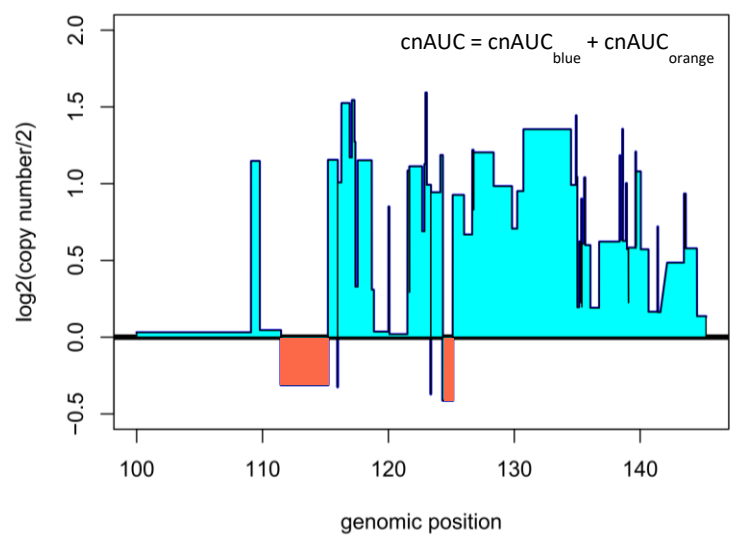

**Figure S2:**

Supplement: Supplementary file 10 — Additional file 10: Figure S2. Pdf format. Calculation of area under the copy number log2 ratio curve (cnAUC). An example of a segmented log2 ratio curve of an amplification SCNA from one tumor is displayed. Due to the nature of the log2 ratio data, the probe specific values fluctuate across the SCNA, with values both above (i.e. gain) and below (i.e. loss) the null reference line of two copies (log2 ratio = 0). To quantify the alteration magnitude for this tumor/SCNA, the cnAUC with respect to the null log2 ratio value of zero was calculated. Area above reference line (blue region) was treated as positive, and area below the line (orange region) was treated as negative. The sum of positive and negative area is the cnAUC of the region for the tumor. [file 12920_2020_765_MOESM10_ESM.pdf]

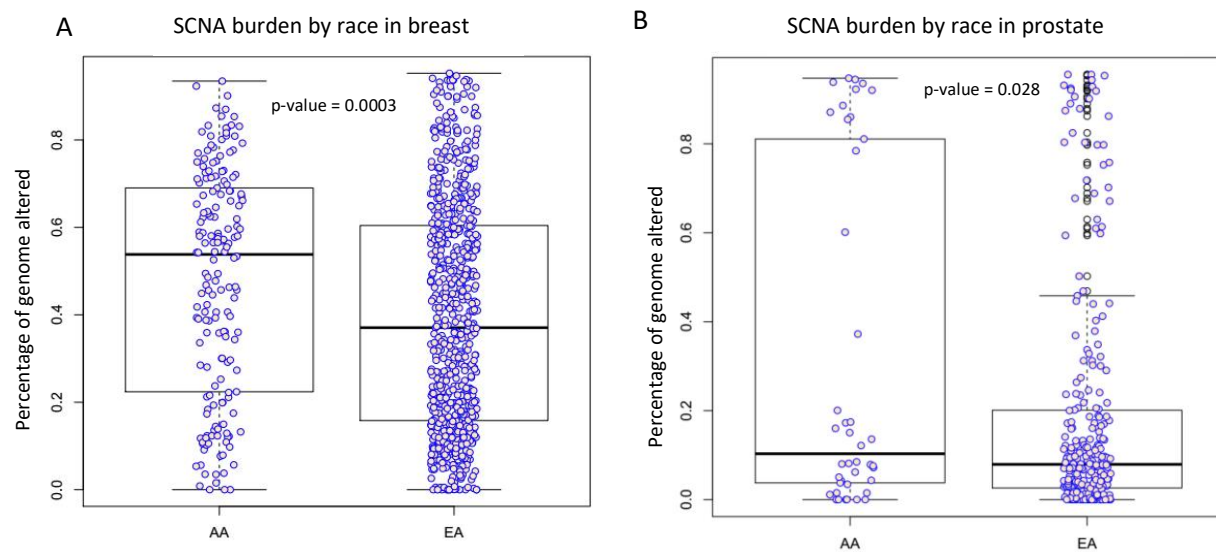

**Figure S3**

Supplement: Supplementary file 11 — Additional file 11: Figure S3. Pdf format. SCNA burden by race in breast and prostate cancer. Distribution of percentage of the genome altered (ie. SCNA burden) by race in A) breast and B) prostate cancer. [file 12920_2020_765_MOESM11_ESM.pdf]

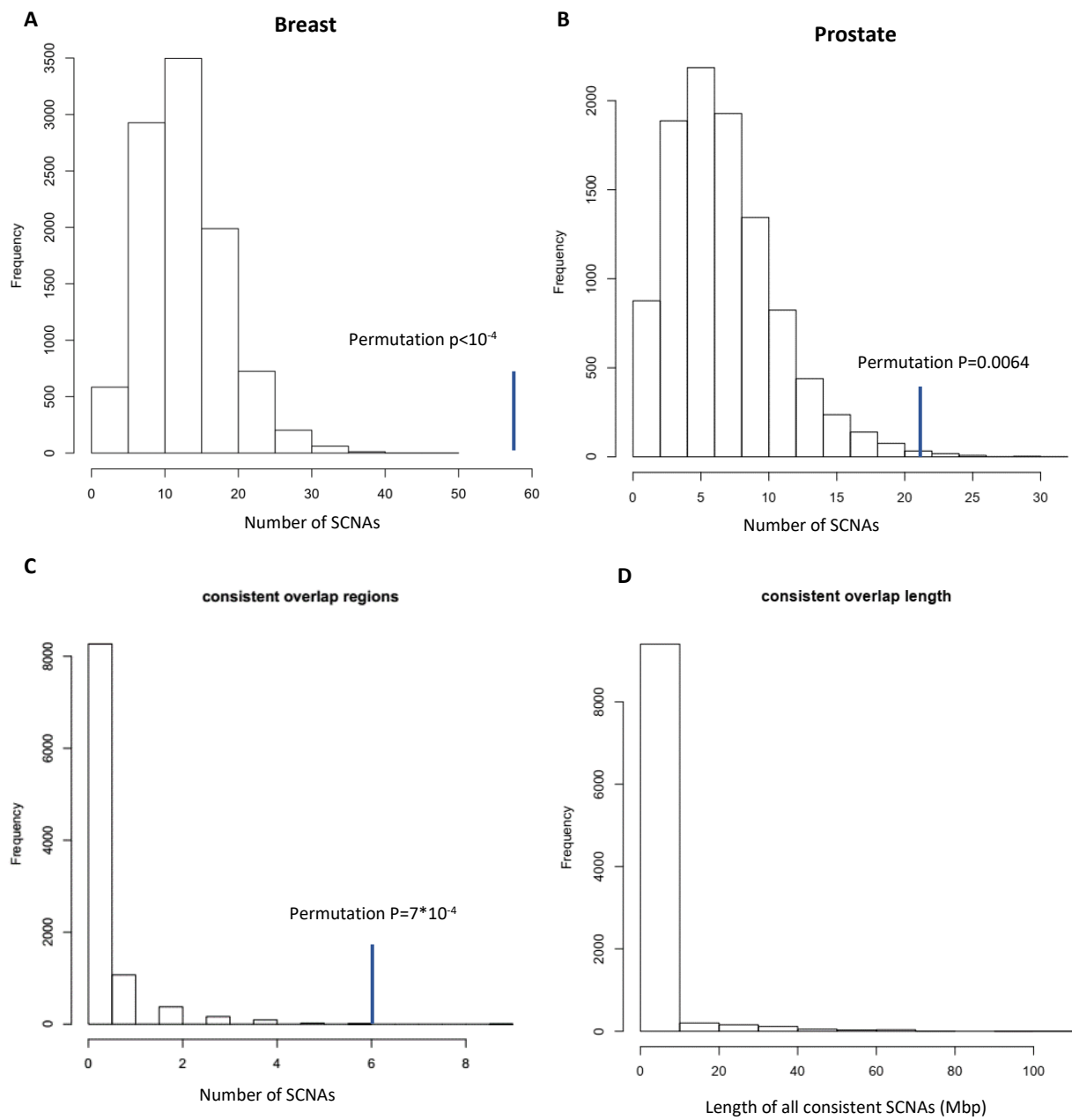

**Figure S4**

Supplement: Supplementary file 12 — Additional file 12: Figure S4. Pdf format. Race permutation results. A) Distribution of the number of SCNA regions with a race-differentiation p-value < 0.1 for each permutation in breast tumors. The mean number is 13.2 SCNAs. B) Distribution of number of SCNA regions with a p-value < 0.1 for each permutation in prostate tumors. The mean number is 7.2 SCNAs. C) Distribution of the number of overlapping race-differentiated SCNAs with consistent direction of changes in breast and prostate tumors. The average number is 0.56 SCNAs. The blue spikes in A-C indicate the number of observed race-differentiated SCNAs in the current study. D) Length distribution of consistent race-differentiated SCNAs. 10,000 permutations were performed to assess significance. [file 12920_2020_765_MOESM12_ESM.pdf]

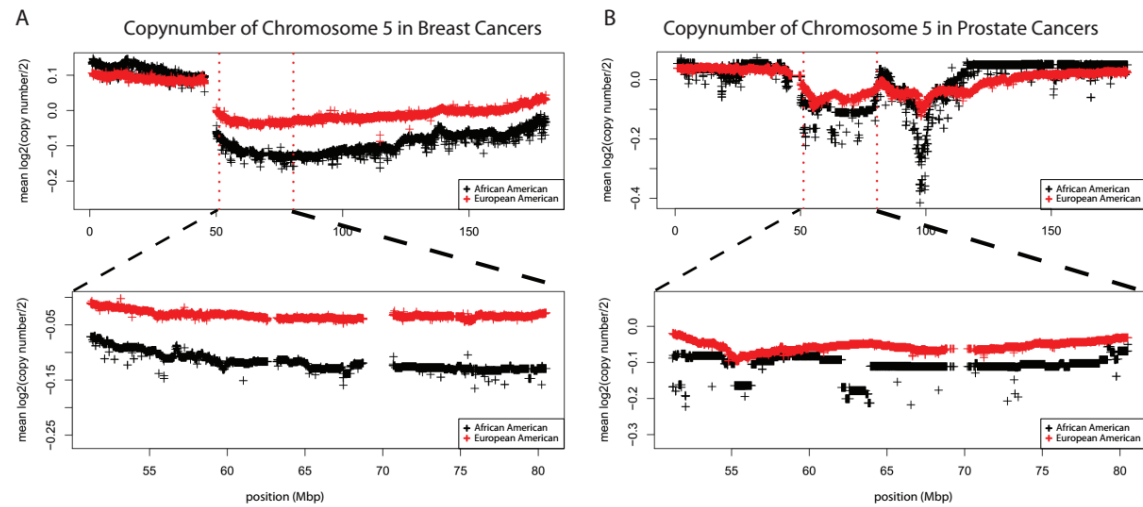

**Figure S5:**

Supplement: Supplementary file 13 — Additional file 13: Figure S5. Pdf format. African American and European American average copy number profiles across chromosome 5q11-q15. A) plots of the breast cancer data and B) plots of the prostate cancer data. In each, the upper panel contains the profile across chromosome 5, and the lower panel highlights the overlapping race-differentiated SCNA region within 5q11-q15 shared by both breast and prostate cancer. [file 12920_2020_765_MOESM13_ESM.pdf]

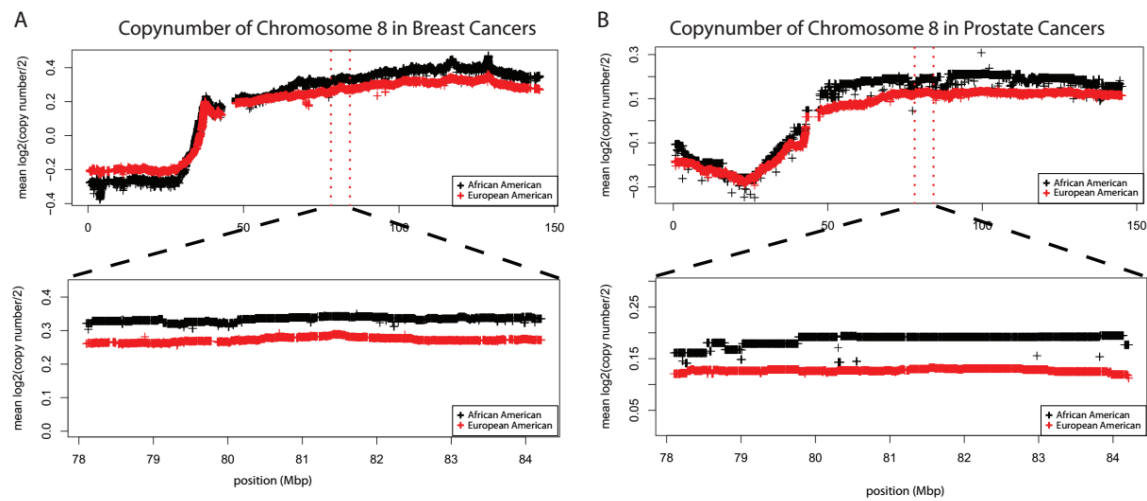

**Figure S6:**

Supplement: Supplementary file 14 — Additional file 14: Figure S6. Pdf format. African American and European American average copy number profiles across chromosome 8q21. A) plots of the breast cancer data, and B) plots of the prostate cancer data. In each, the upper panel contains the profile across chromosome 8, and the lower panel highlights the overlapping race-differentiated SCNA region within 8q21 shared by both breast and prostate cancer. [file 12920_2020_765_MOESM14_ESM.pdf]

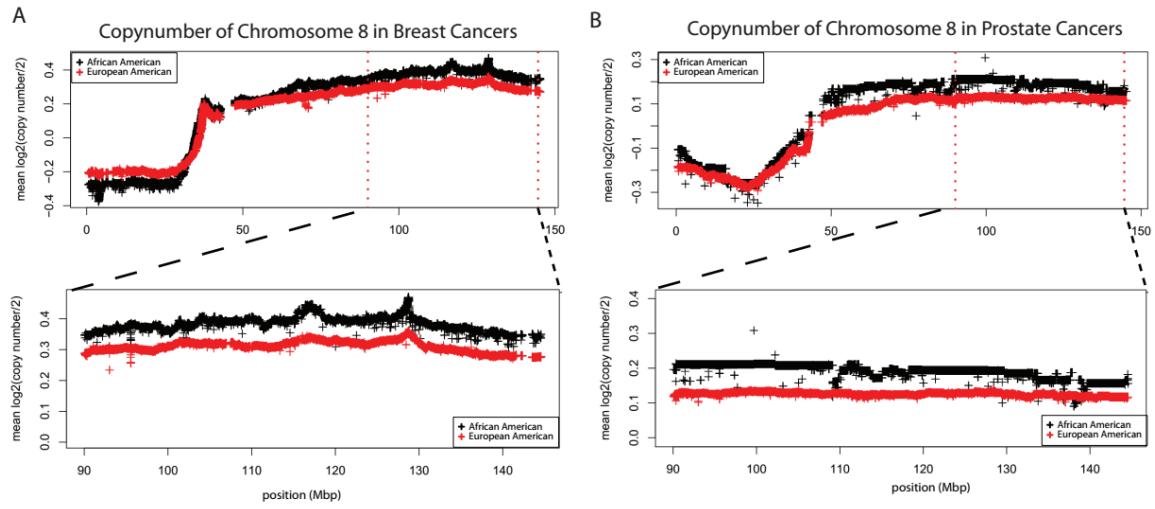

**Figure S7:**

Supplement: Supplementary file 15 — Additional file 15: Figure S7. Pdf format. African American and European American average copy number profiles across chromosome 8q21-q24. A) plots of the breast cancer data, and B) plots of the prostate cancer data. In each, the upper panel contains the profile across chromosome 8, and the lower panel highlights the overlapping race-differentiated SCNA region within 8q21-q24 shared by both breast and prostate cancer. [file 12920_2020_765_MOESM15_ESM.pdf]

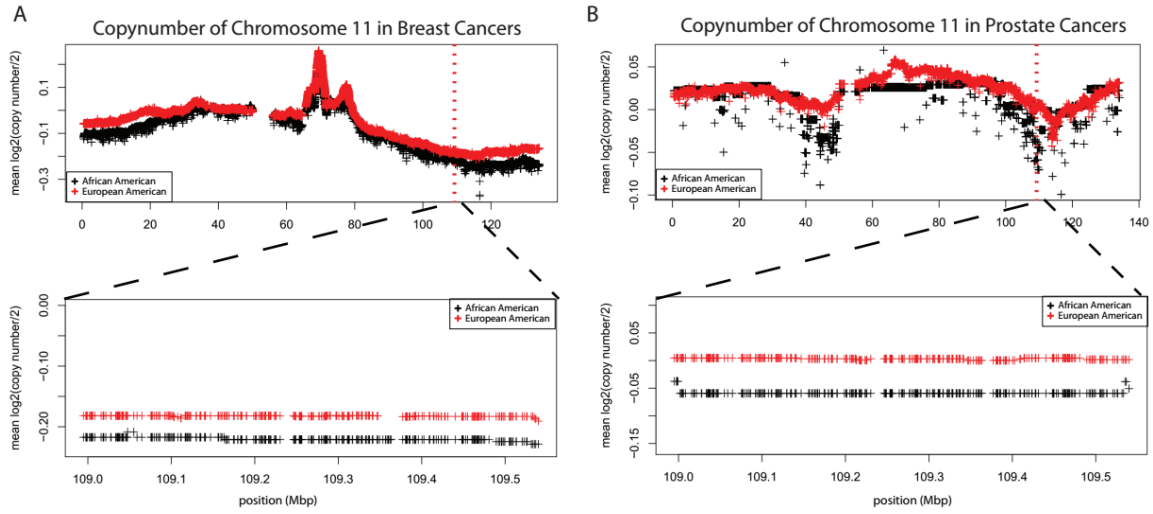

**Figure S8:**

Supplement: Supplementary file 16 — Additional file 16: Figure S8. Pdf format. African American and European American average copy number profiles across chromosome 11q22. A) plots of the breast cancer data, and B) plots of the prostate cancer data. In each, the upper panel contains the profile across chromosome 11, and the lower panel highlights the overlapping race-differentiated SCNA region within 11q22 shared by both breast and prostate cancer. [file 12920_2020_765_MOESM16_ESM.pdf]

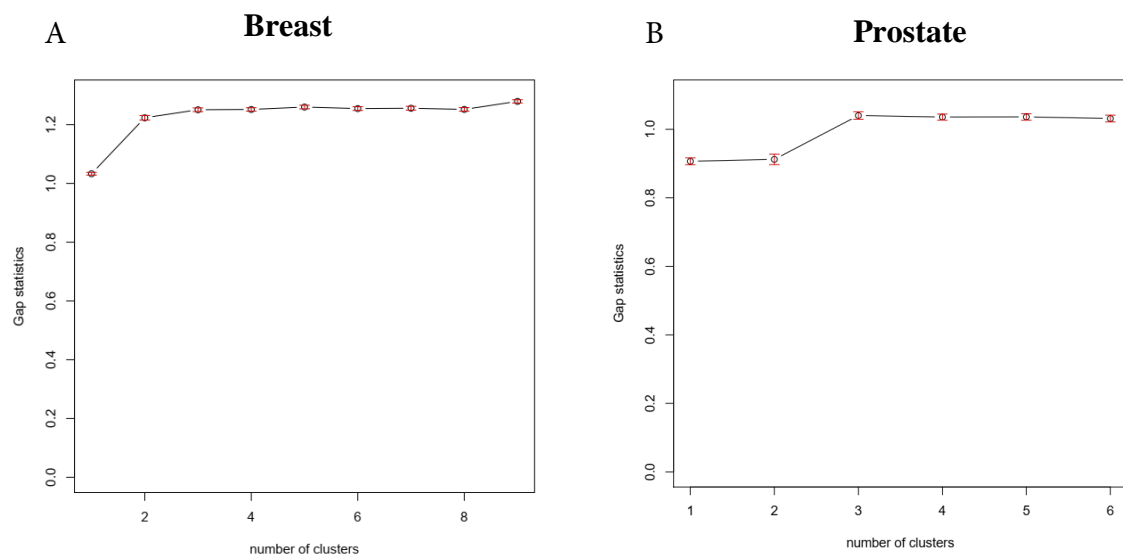

**Figure S9:**

Supplement: Supplementary file 17 — Additional file 17: Figure S9.Pdf format. Gap statistic plots for copy number clustering in A) breast and B) prostate cancers. The point at which the gap statistic first reaches a maximum indicates the most likely number of patient groups (clusters) within the cancer types. For both breast and prostate cancer, this occurred at a value of three. [file 12920_2020_765_MOESM17_ESM.pdf]

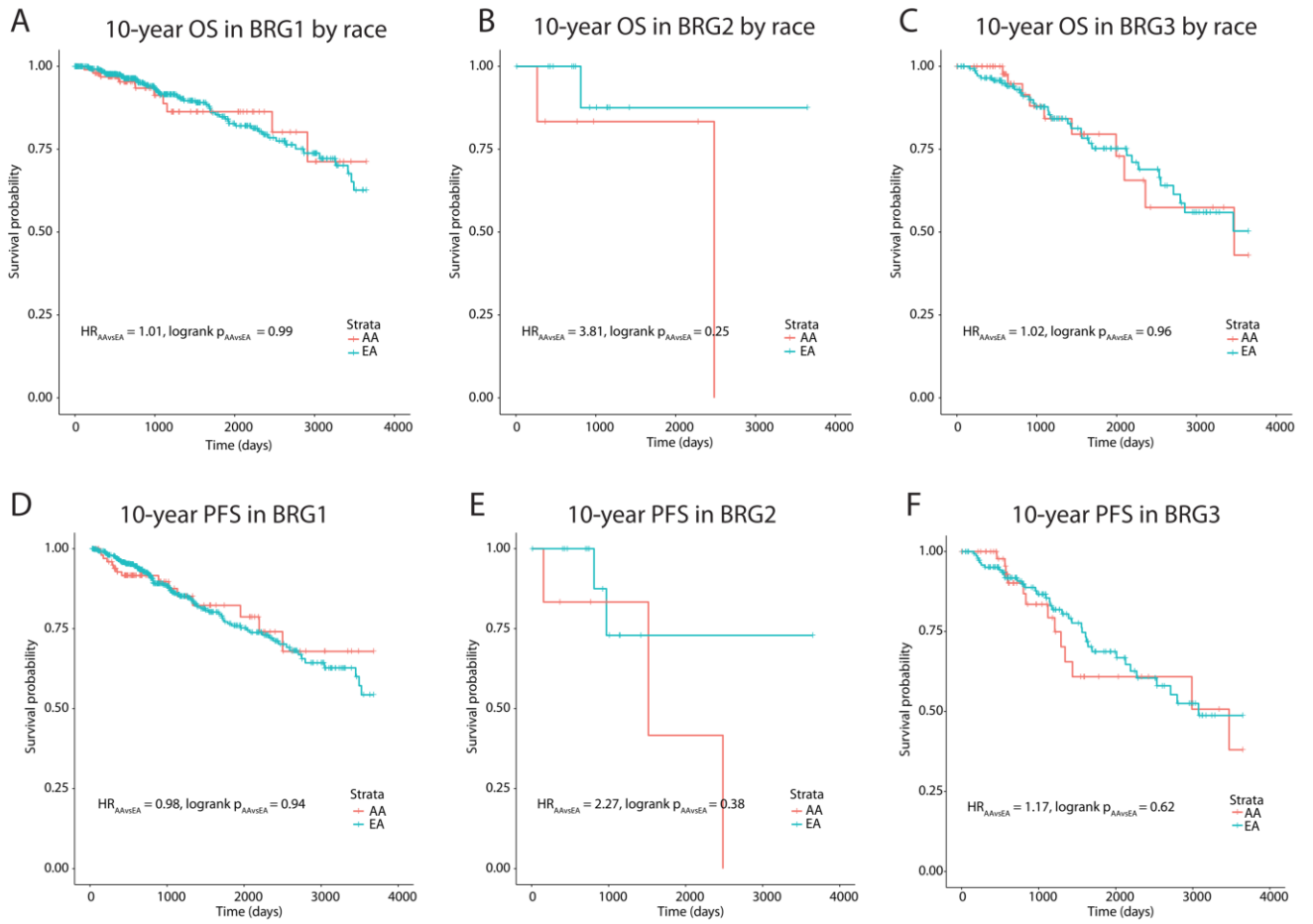

**Figure S10:**

Supplement: Supplementary file 18 — Additional file 18: Figure S10. Pdf format. Breast cancer survival for each SCNA-defined breast cancer patient group (BRG) by race. A-C) Kaplan-Meier 10-year overall survival (OS) curves for each BRG, by race. D-F) Kaplan-Meier 10-year progression-free survival (PFS) curves for each BRG, by race. [file 12920_2020_765_MOESM18_ESM.pdf]

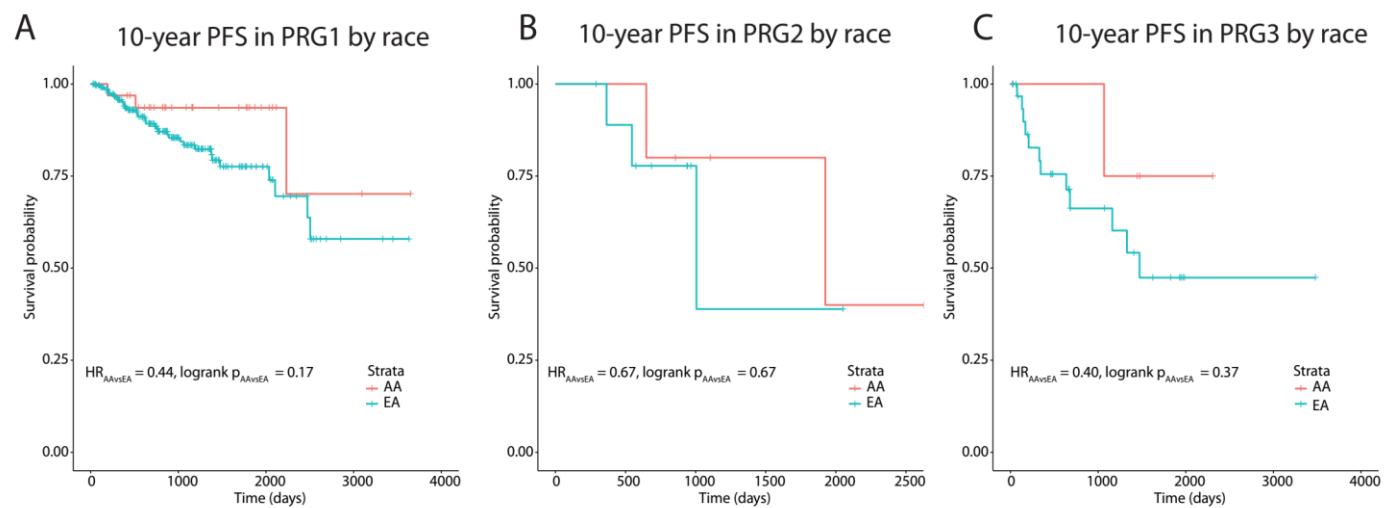

**Figure S11:**

Supplement: Supplementary file 19 — Additional file 19: Figure S11. Pdf format. Prostate cancer progression-free survival (PFS) for each SCNA-defined prostate cancer patient group (PRGs) by race. A-C) Kaplan-Meier 10-year PFS curves for each PRG, by race. [file 12920_2020_765_MOESM19_ESM.pdf]
